# Impact of Nurse‐Driven Analgesia and Sedation Protocols on Medication Exposure and Withdrawal in Critically Ill Children: A Systematic Review

**DOI:** 10.1111/nicc.70051

**Published:** 2025-05-29

**Authors:** Laura Schianchi, Julia Harris

**Affiliations:** ^1^ Paediatric and Adult Congenital Heart Disease Centre IRCCS Policlinico San Donato San Donato Milanese (MI) Italy; ^2^ Department of Children's Nursing London South Bank University London UK

**Keywords:** analgesia and sedation protocol, benzodiazepines, iatrogenic withdrawal syndrome (IWS), nurse‐driven protocol, paediatric intensive care unit (PICU)

## Abstract

**Background:**

The administration of analgesia and sedation is essential for patients admitted to the paediatric intensive care unit (PICU). However, over‐sedation can cause side effects, including iatrogenic withdrawal syndrome (IWS). The use of nurse‐led analgesia and sedation protocols may improve patient outcomes.

**Aim:**

The primary aim of this systematic review was to determine whether the use of such protocols can reduce opioid and benzodiazepine doses. Secondary outcome measures included documentation of pain and sedation scores, incidence of IWS, duration of mechanical ventilation (MV) and PICU/hospital length of stay (LOS).

**Study Design:**

A systematic review of the literature was conducted, searching several databases, including CINAHL, MEDLINE, Academic Search Complete and Cochrane Library. Pertinent articles were selected according to pre‐determined eligibility criteria. The internal validity of included studies was assessed using validated critical appraisal tools for quantitative research from the Cochrane Library. Narrative synthesis was utilised for data analysis due to the heterogeneity of study characteristics.

**Results:**

Nurse‐led protocolised sedation significantly reduced the administered doses of benzodiazepines and the incidence of IWS. Moreover, the use of protocols significantly improved the documentation of pain and sedation scores across included studies. No significant difference in opioid use, duration of MV, and PICU/hospital LOS has been found. However, sub‐group analyses for duration of MV and PICU/hospital LOS showed positive results in older children and those post‐cardiac surgery.

**Conclusions:**

Nurse‐driven analgesia and sedation protocols can reduce over‐sedation and IWS in critically ill children. Further studies should explore the use of protocols in patient sub‐groups where positive results have been reported.

**Relevance to Clinical Practice:**

Nurse‐led analgesia and sedation protocols in PICU can improve outcomes and reduce costs. Effective implementation requires training and audits to boost nurses' confidence and autonomy.

**Summary**

- What is known about the topic:
  ○ Pain and sedation management in PICU is challenging, often leading to over‐sedation and associated risks like prolonged ventilation, drug tolerance, withdrawal, delirium and neurotoxicity.
  ○ National and international guidelines exist, but clinical practice remains inconsistent across settings.
  ○ Nurse‐led analgesia and sedation protocols aim to standardise care but have shown limited success in reducing mechanical ventilation duration.
- What this paper adds:
  ○ Nurse‐led protocols in PICU may reduce the use of analgesics and sedatives, particularly benzodiazepines, and help minimise drug tolerance and withdrawal symptoms.
  ○ These protocols promote standardised, multidisciplinary care, involving bedside nurses in decision‐making.
  ○ They may also reduce ventilation time in older or lower‐risk children and shorten PICU/hospital stays in cardiac patients with simple defects, improving surgical outcomes.

## 1 Introduction

Paediatric patients with critical illnesses admitted to the paediatric intensive care unit (PICU) require analgesic and sedative medications for invasive procedures, such as surgical interventions, intubation and drain insertion. These medications are also essential for patient‐ventilator synchrony and postoperative haemodynamic stability [1]. Effective pain management improves clinical and psychological outcomes, while untreated pain and anxiety can cause complications, including inadequate ventilation and unplanned extubation [2]. Achieving optimal sedation, where patients are comfortably asleep yet easily arousable, is challenging [3]. Over‐sedation may prolong mechanical ventilation (MV) and increase medication tolerance, leading to iatrogenic withdrawal syndrome (IWS), delirium and neurotoxicity [4]. Current practices aim to minimise sedation, facilitate early extubation and ensure adequate pain management [5]. Despite clinical guidelines, substantial variability in practice remains [6, 7]. Analgesia and sedation protocols may reduce variability and improve patient outcomes [8]. Such protocols are formally approved written algorithms that prioritise validated pain assessments and patient‐specific sedation targets [9].

## 2 Background

Bedside nurses regularly assess pain and sedation, allowing them to take immediate action rather than waiting for doctors to make rounds, which could delay timely interventions, particularly with weaning. A nurse‐driven analgesia and sedation protocol, prescribed by doctors and implemented by nurses, could improve inter‐professional collaboration and patient safety [10, 11]. Although current guidelines recommend protocolised sedation for all ventilated paediatric patients [6, 7], evidence remains inconsistent [12, 13]. Individual adult clinical trials demonstrate reductions in duration of MV, ICU length of stay (LOS) and mortality [14, 15, 16]. However, a systematic review and meta‐analysis failed to demonstrate the superiority of protocols in mechanically ventilated paediatric and adult patients [13]. Further studies are needed to more effectively inform clinical practice. In this regard, a new systematic review will be conducted to explore a broad range of outcomes. By considering multiple outcomes, this study aims to provide a comprehensive understanding of the intervention's effects and ensure the findings are applicable to diverse populations and settings. This approach will not only enhance decision‐making but also identify gaps in the current evidence and guide future research efforts.

## 3 Aim and Objectives

This systematic review aims to evaluate the impact of nurse‐driven analgesia and sedation protocols on reducing exposure to opioids and benzodiazepines. Secondary objectives include evaluating improvements in pain and sedation score documentation, incidence of IWS and the duration of MV, PICU and hospital stays.

## 4 Design and Methods

### 4.1 Research Question

This systematic review was carried out in accordance with the PRISMA statement and the guidelines outlined in the Cochrane Handbook for Systematic Reviews of Interventions [17, 18]. The research question was developed using the PICO framework, as detailed below:

- Population: critically ill paediatric patients (< 18 years) admitted to PICU.
- Intervention: nurse‐driven analgesia and sedation protocol.
- Comparison: standard care.
- Outcome: opioid and benzodiazepine use.

### 4.2 Eligibility Criteria

Eligibility criteria were pre‐determined to guide the article selection process, according to the PICO framework. The inclusion criteria for this review were studies conducted in PICUs involving patients under 18 years of age, utilising nurse‐driven analgesia and/or sedation protocols, and measuring opioid and/or benzodiazepine exposure. Studies had to be peer‐reviewed, published in the last 10 years, and written in English. Exclusion criteria included studies conducted in adult or neonatal intensive care units (AICUs or NICUs) and those exclusively involving patients on extracorporeal membrane oxygenation (ECMO). Due to the paucity of RCTs, no restrictions were imposed according to the study design of selected articles.

### 4.3 Search Strategy

CINAHL (Cumulative Index to Nursing and Allied Health Literature), MEDLINE (Medical Literature Analysis and Retrieval System Online), Academic Search Complete and the Cochrane Library were systematically searched, utilising the following search terms: nurse‐driven, nurse‐directed, nurse‐led, nurse‐implemented, analgesia, pain relief, pain management, sedation, opioids, morphine, benzodiazepines, midazolam, paediatric intensive care unit, pediatric intensive care unit, PICU, paediatric critical care unit, pediatric critical care unit. The full search strategy is provided as Figure S1.

### 4.4 Study Selection Process

The study selection process involved two reviewers who independently screened the studies based on predefined eligibility criteria. Any disagreements between the reviewers were resolved through discussion, and if consensus could not be reached, a third‐party adjudicator was consulted to make the final decision. EndNote 20 was utilised to manage and organise references efficiently. This ensured consistency and rigour throughout the study selection process, maintaining transparency and reducing the risk of bias.

### 4.5 Critical Appraisal

The risk of bias in the included studies was assessed with validated tools from the Cochrane Library. For cluster‐randomised trials, we utilised the Revised Cochrane risk‐of‐bias tool for cluster‐randomised trials (RoB 2 CRT), which evaluates biases across domains such as randomisation, deviations from intended interventions, missing outcome data, measurement of the outcome, and selection of the reported results. For non‐randomised studies, we applied the Risk of Bias In Non‐randomised Studies—of Interventions, Version 2 (ROBINS‐I V2), a structured tool designed to assess biases related to confounding, selection, classification of interventions, deviations from intended interventions, missing data, measurement of outcomes and selection of reported results. Each study was independently assessed by two reviewers, with discrepancies resolved through discussion or consultation with a third reviewer. A summary table was constructed to present the risk‐of‐bias judgements across all included studies, ensuring transparency and methodological rigor in the assessment process.

### 4.6 Data Extraction

A pre‐specified data extraction table was adapted from the data extraction guidelines published by the Joanna Briggs Institute (JBI) [19] and structured according to the key components of the PICO framework. The extracted variables included study design, country, sample size, intervention protocols, pain and sedation assessment tools, withdrawal assessment tools, and relevant findings. Data were independently extracted from the full texts of eligible studies and cross‐verified to minimise discrepancies. Where applicable, pre‐ and post‐implementation periods were documented to facilitate comparative analysis. Standardised pain, sedation, and withdrawal assessment scales, as reported in the original studies, were retained to ensure methodological rigour. Any discrepancies in data interpretation were resolved through consensus discussions. The final extracted data were systematically organised into a structured tabular format to enable clear cross‐study comparison and synthesis.

### 4.7 Data Analysis

Given the significant heterogeneity in the included studies, including differences in study populations, interventions and outcome measures, a meta‐analysis was not conducted. The studies varied substantially in terms of the drugs and dosages used, as well as the units employed to express reductions in opioid and benzodiazepine use. Furthermore, the methods for assessing withdrawal symptoms differed across studies, making it impractical to pool the data meaningfully. While random‐effects models and subgroup analyses are commonly applied to address heterogeneity, the extent of variability in both study characteristics and outcomes would have undermined the robustness and validity of any pooled estimates, potentially leading to misleading conclusions. As such, the results of this systematic review have been synthesized in a narrative format. To enhance understanding and facilitate comparisons across studies, we have included bar charts, which highlight key findings. This approach ensures a clearer presentation of the data while maintaining the methodological rigour of the review.

## 5 Results

**FIGURE 1:**
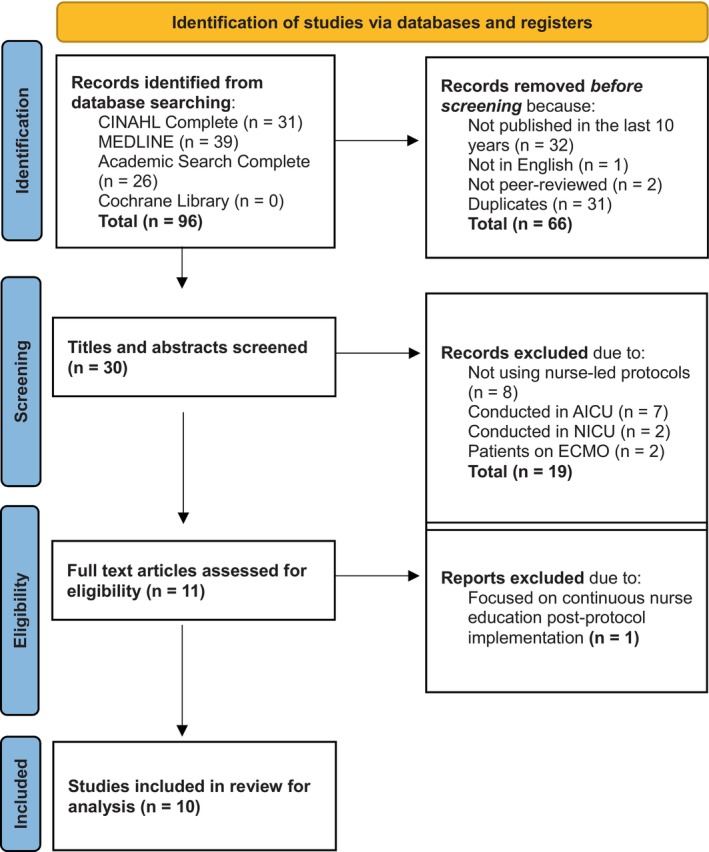
PRISMA flow diagram. AICU: adult intensive care unit, ECMO: extracorporeal membrane oxygenation, NICU: neonatal intensive care unit.

A total of 10 articles were included in the review for analysis. The study selection process is displayed in Figure 1.

### 5.1 Results of Critical Appraisal

The included studies are all quantitative and interventional, consisting of one RCT and nine single‐arm before‐and‐after studies without control groups. While such non‐randomised designs are often suitable for evaluating new clinical protocols, they pose challenges related to baseline comparability and confounding [20, 21]. The risk‐of‐bias assessment, conducted using the RoB 2 and ROBINS‐I tools, found that the RESTORE trial [22] had a moderate risk of bias due to recruitment and reporting concerns, while non‐randomised studies exhibited varying levels of bias. Cardiac‐RESTORE [23] had a moderate overall risk, whereas Michel et al. (2020) [24] and Magner et al. (2020) [25] were deemed to have a critical risk of bias, particularly due to confounding and selective reporting. Despite some studies having low bias in certain domains, none were entirely free from bias, and most had an overall serious risk, highlighting the need for cautious interpretation and improved methodologies in future research. Summary results are available in Tables 1 and 2, with detailed assessments provided in Supporting Information: Documents 1–10.

**TABLE 1:** Risk of bias assessment of randomised studies.

Study	Randomisation (1a)	Recruitment of participants (1b)	Deviations from intended interventions (2)	Missing data (3)	Measurement of outcomes (4)	Selection of reported results (5)	Overall risk of bias
RESTORE Trial—Curley et al. (2015)	Low	Some concerns	Low	Low	Some concerns	Some concerns	Some concerns

**TABLE 2:** Risk of bias assessment of non‐randomised studies.

Study	Confounding (1)	Classification of interventions (2)	Selection of participants (3)	Deviations from intended interventions (4)	Missing data (5)	Measurement of outcomes (6)	Selection of reported results (7)	Overall risk of bias
Cardiac‐RESTORE—Lincoln et al. (2020)	Low except for concerns about uncontrolled confounding factors	Low	Low	Low	Low	Moderate	Low	Moderate
Dreyfus et al. (2017)	Low risk of bias except for concerns about uncontrolled confounders	Low	Low	Low	Low	Serious	Moderate	Serious
Gaillard‐Le Roux et al. (2017)	Moderate	Moderate	Moderate	Moderate	Low	Serious	Serious	Serious
Hanser et al. (2020)	Serious	Low	Low	Moderate	Low	Serious	Moderate	Serious
Larson and McKeever (2018)	Serious	Low	Serious	Moderate	Low	Serious	Serious	Serious
Neunhoeffer et al. (2015)	Serious	Low	Low	Moderate	Low	Serious	Moderate	Serious
Neunhoeffer et al. (2017)	Serious	Low	Low	Moderate	Low	Serious	Serious	Serious
Magner et al. (2020)	Low except for concerns about uncontrolled confounding	Low	Low	Low	Low	Serious	Critical	Critical
Michel et al. (2020)	Critical	Moderate	Low	Moderate	Low	Serious	Moderate	Critical

### 5.2 Study Characteristics

The primary characteristics of the included studies are summarised in Table 3. The studies were conducted in countries such as the United States, Ireland, France, Germany and Australia. Sample sizes varied from 65 to 2449 paediatric patients, encompassing both medical and post‐surgical populations. The largest studies were conducted in the United States, notably the RESTORE trial [22] (2015), a cluster‐randomised trial involving 31 PICUs and the cardiac‐RESTORE project [23] (2020), a quality improvement initiative with a pre–post interval measurement framework. The cardiac‐RESTORE protocol was adapted from the original RESTORE protocol, incorporating a fast‐track section designed specifically to manage the immediate postoperative period for cardiac surgical patients. Most protocols emphasised nurse‐led, goal‐directed sedation and analgesia titration, guided by validated tools, such as the COMFORT‐Behaviour Scale (COMFORT‐B), Nurse Interpretation of Sedation Scale (NISS) and State Behavioural Scale (SBS) for sedation, as well as the Face, Legs, Activity, Cry, Consolability Scale (FLACC) or Numerical Rating Scale (NRS) for pain assessment. Withdrawal symptoms were predominantly evaluated using the Withdrawal Assessment Tool‐1 (WAT‐1) and the Sophia Observational withdrawal Symptoms Scale (SOS). Implementation strategies focused on multidisciplinary training, the use of standardised protocols, and weaning guidelines, which were often adapted to the specific resources and patient demographics of each study site.

**TABLE 3:** Summary table of study characteristics.

Authors and year	Design	Country	Sample	Protocol	Pain and sedation assessment tools	Withdrawal assessment tools	Relevant findings
Curley et al., 2015 [23]	Cluster‐randomized trial conducted in 31 U.S. PICUs.	United States	2449 patients with acute respiratory failure.	**RESTORE protocol** Approach: nurse‐implemented, goal‐directed algorithm with interprofessional team training. Phases of illness: acute, titration, weaning. Arousal assessments and ERT daily. Sedative agents: primary: morphine, midazolam (fentanyl for specific conditions). Secondary: dexmedetomidine, propofol, clonidine, pentobarbital, ketamine. Sedation adjustments: titration every 8 h based on illness phase and SBS. Training: multi‐disciplinary team trained in algorithm, including nurses, physicians, and respiratory therapists. Control: control units managed sedation without a protocol.	Pain: pain scores (specific scales not reported). Sedation: SBS to set sedation targets.	WAT–1 used to assess withdrawal symptoms. Clonidine for management, methadone if symptoms persist.	**Sedation Strategy:** the intervention group used morphine and midazolam as primary agents, while the control group predominantly used fentanyl, midazolam, and lorazepam, along with dexmedetomidine. **Opioid Exposure** Mean daily dose (mg/kg): intervention: 1.3 (0.7–2.6) vs control: 1.7 (0.8–2.9). *p‐value* = 0.13. Peak daily dose (mg/kg): intervention: 3.3 (1.6–6.1) vs control: 4.0 (2.0–7.0). *p‐value* = 0.16. Cumulative dose (mg/kg): intervention: 13.7 (5.3–38.3) vs control: 17.7 (5.3–52.9). *p‐value* = 0.10. Exposure days (days): intervention: 9 (5–15) vs control: 10 (4–21). *p‐value* = 0.01*. **Benzodiazepine Exposure** Mean daily dose (mg/kg): intervention: 1.3 (0.6–2.7) vs control: 1.3 (0.6–2.5). *p‐value* = 0.22. Peak daily dose (mg/kg): intervention: 2.9 (1.5–6.0) vs control: 3.2 (1.5–6.7). *p‐value* = 0.77.
							Cumulative dose (mg/kg): intervention: 14.0 (5.1–41.5) vs control: 13.6 (4.3–42.4). *p‐value* = 0.54. **Incidence of Clinically Significant Iatrogenic Withdrawal:** intervention: 12% (149 patients) vs control: 9% (114 patients). *p‐value* = 0.80. No effect size provided; no significant difference observed. **Median Duration of Mechanical Ventilation (Days):** intervention: 6.5 days (IQR: 4.1–11.2) vs control: 6.5 days (IQR: 3.7–12.1). *p‐value* = 0.61. Adjusted analysis accounting for confounders (age, PRISM III–12 score, POPC > 1): *p‐value* = 0.95. **PICU Length of Stay:** intervention: 9.6 days (IQR: 6.2–15.6) vs control: 9.6 days (IQR: 5.7–16.6). *p‐value* = 0.61. **Hospital Length of Stay:** intervention: 14 days (IQR: 9–24) vs control: 16 days (IQR: 9–29). *p‐value* = 0.20.
Lincoln et al., 2020 [24]	Quality improvement project with a pre‐post interval measurement plan conducted in a 31‐bed paediatric CICU. Time points: Pre‐implementation interval: patients hospitalized between December 2014 and February 2015, before cardiac‐RESTORE was introduced. Post‐implementation interval 1: patients from May 2015 to July 2015, representing the initial period after the cardiac‐RESTORE protocol was introduced.	United States	1243 post‐cardiac surgery patients	**Cardiac‐RESTORE** Replication of the previously published RESTORE protocol with the addition of a fast–track section to manage the immediate postoperative period in cardiac surgical patients. Daily team discussion of the patient's trajectory of illness (acute, titration, or weaning phase) Daily prescription of a sedation target per phase of illness using the SBS. Arousal assessments if the patient is too sedate (reducing sedative infusions by 50% until the patient's SBS matches the sedation target). Titration of sedatives to achieve the prescribed sedation target at least every 8 hours.	Pain: FLACC (nonverbal children 0–6 years old) NRS (6 years old or older) Wong‐Baker Faces Pain Scale (3 years old or older) Sedation: SBS	Documentation of WAT‐1 scores systemically implemented with cardiac‐RESTORE.	**Opioid Reduction** *Morphine* Cumulative Dose (mg/kg/visit): median dose reduced from 1.12 (0.28–5.92) to 0.60 (0.25–2.23). *p‐value* = 0.008*. Average Dose (mg/kg/day): median dose reduced from 0.21 (0.10–0.62) to 0.15 (0.07–0.38). *p‐value* = 0.005*. Peak Dose (mg/kg/day): Median dose reduced from 0.51 (0.15–2.15) to 0.29 (0.15–0.95). *p‐value* = 0.007*. *Fentanyl* Cumulative Dose (mcg/kg/visit): median dose reduced from 8.97 (1.40–36.6) to 2.73 (0.39–12.2). *p‐value* < 0.001*. Average Dose (mcg/kg/day): median dose reduced from 1.15 (0.46–3.03) to 0.93 (0.31–2.41). *p‐value* = 0.023*. Peak Dose (mcg/kg/day): median dose reduced from 2.95 (0.94–6.97) to 1.99 (0.39–5.16). *p‐value* = 0.002*.
	Post‐implementation interval 2: September 2015 to February 2016, continuing the evaluation after the initial implementation phase. Post‐implementation interval 3: final cohort of patients admitted from September 2016 to February 2017, further assessing the sustainability and effects of cardiac‐RESTORE.			Discontinuation of opioids and benzodiazepines when no longer necessary (if opioid/sedative exposure < 5 days) or weaned per target.			**Benzodiazepine Reduction** *Midazolam* Cumulative Dose (mg/kg/visit): median dose reduced from 0.60 (0.12–3.68) to 0.25 (0.08–1.09) post–RESTORE. *p‐value* < 0.001*. Average Dose (mg/kg/day): median dose reduced from 0.13 (0.05–0.50) to 0.08 (0.04–0.23). *p‐value* < 0.001*. Peak Dose (mg/kg/day): median dose reduced from 0.32 (0.08–1.45) to 0.15 (0.05–0.52). *p‐value* < 0.001*. *Lorazepam* Cumulative Dose (mg/kg/visit): median dose reduced from 0.32 (0.15–9.09) to 0.17 (0.07–0.93). *p‐value* = 0.007*. Average Dose (mg/kg/day): median dose reduced from 0.17 (0.07–0.61) to 0.09 (0.06–0.24). *p‐value* = 0.011*. Peak Dose (mg/kg/day): median dose reduced from 0.17 (0.08–1.13) to 0.14 (0.07–0.38). *p‐value* = 0.010*. **Duration of Mechanical Ventilation (Hours)** Median duration reduced from 16 h (IQR: 5–47) pre‐RESTORE to: 11 h (IQR: 4–29) in post–RESTORE 1, 22 h (IQR: 9–75) in post–RESTORE 2 and 18 h (IQR: 8–60) in post‐RESTORE 3. *p‐value* < 0.001*.
							**Postoperative CICU Length of Stay (Days)** Median duration reduced from 3 days (IQR: 2–8) pre‐RESTORE to 2 days (IQR: 1–5) in all post–RESTORE time intervals. *p‐value* < 0.001*.
Magner et al., 2020 [25]	Retrospective before‐and–after study at a 23‐bed PICU.	Ireland	125 post‐cardiac surgery patients	Guideline development & training: PICU Pain and Sedation Group developed guidelines. 6‐month education campaign with mandatory staff training on new guidelines. Analgesia and sedation guidelines: Initial post‐operative assessment using COMFORT‐B scale. Morphine prescription with specified infusion rates, bolus doses, and transition to oral morphine for weaning. Acetaminophen administration every 6 h initially, then oral once possible. NSAIDs recommended if not contraindicated. Sedation: IV midazolam or enteral chloral hydrate if needed.	Pain: NRS Sedation: COMFORT‐B	Not assessed	**Total Morphine Administered (72 h):** before: median 987 mcg/kg (IQR 730–1637) vs after: median 949 mcg/kg (IQR 731–1314). *p‐value* = 0.46. **Duration of Mechanical Ventilation (Hours)** **Patients with Trisomy 21:** before: median 34 h vs after: median 25.1 hours. *p‐value* = 0.012*. **Patients without Trisomy 21:** before: median 30.5 h vs after: median 25.7 hours. *p‐value* = 0.07. **PICU Length of Stay (Days):** before: median 3.5 days (IQR 1.9–6.3) vs after: median 2.8 days (IQR 1.9–4.7). *p‐value* = 0.28. COMFORT‐B and NRS assessments conducted only after the intervention.
				Weaning: Morphine weaning with oral transition and clonidine as an adjunct.			
Dreyfus et al., 2017 [26]	Before and after protocol implementation study. Time points: 12‐month pre‐implementation period between January 2013 and December 2013. 11–month post‐implementation period between May 2014 and March 2015. The post‐implementation data collection period started 4 months after the implementation of the protocol in January 2014. During this 4‐month lag, the protocol was introduced and explained to all the nurses and medical staff and no data was collected.	France	200 medical and surgical patients, MV for > 24 hours.	Training: Over four months, training courses were conducted, with sessions focused on sedation, analgesia, COMFORT‐B scale evaluation, and protocol use. Clinical scenarios and questionnaires were also used during training. Sedation management: physicians initially prescribed doses, and nurses adjusted them according to the protocol aiming for optimal sedation (COMFORT‐B score 11–17). Adjustment process: if the score was above 17 (insufficient sedation), nurses first increased the sufentanil dose, then, if necessary, adjusted hypnotics (midazolam or ketamine). If the score fell below 11 (oversedation), drugs were reduced alternately every hour.	Pain and sedation: COMFORT‐B.	WAT–1 with a score of ≥ 3 indicating withdrawal symptoms.	**Opioid Exposure** *Sufentanil* Daily dose (mcg/kg/day): Pre‐implementation: 0.19 (SD = 0.31) vs post‐implementation –0.21 (SD = 0.44). *p‐value* = 0.668. Duration of administration (hours): pre‐implementation: –3.31 (SD = 4.79) vs post‐implementation: –1.32 (SD = 6.78). *p‐value* = 0.855. **Benzodiazepine Exposure** *Midazolam* Daily dose (mg/kg/day): pre‐implementation: −0.12 (SD = 0.18) vs post‐implementation: 0.02 (0.25). *p‐value* = 0.944. Duration of administration (hours): Pre‐implementation: 0.27 (SD = 3.59) vs post‐implementation: −3.39 (SD = 5.07). *p‐value* = 0.541. **Reduction in Withdrawal Symptoms** Pre‐implementation: 24/104 = 23% incidence vs post‐implementation: 13/93 = 14% incidence. *p‐value*: 0.103.
				Drug dosing: maximum doses for drugs (midazolam, ketamine, sufentanil) were set. If these doses were reached, the physician was informed. Bolus dosing: In addition to continuous infusion, hourly boluses were allowed, especially during nursing care.			**Duration of Mechanical Ventilation (Days)** Pre‐implementation: mean = 8.3 (SD = 7.3) vs post‐implementation: mean = 6.6 (SD = 5.6). Overall trend before implementation: −0.35 (0.42). *p‐value*: 0.450. Immediate change after implementation: 0.17 (1.64). *p‐value* = 0.923. Change in trend after implementation: −0.05 (0.60). *p‐value* = 0.933. **PICU Length of Stay (Days)** Pre‐implementation: mean = 9.0 (SD = 5.0–15.2) vs post‐implementation: mean = 9.8 (SD = 4.9–14.5). Overall trend before implementation: −0.71 (0.90). *p‐value* = 0.476. Immediate change after implementation: 6.56 (3.49) days. *p‐value* = 0.134. Change in trend after implementation: −1.07 (1.28). *p‐value* = 0.450. **Documentation of Pain/Sedation Scores** Frequency of COMFORT‐B Assessments: Pre‐implementation: mean = 3.9 assessments/day (SD = 2.5) vs post‐implementation: mean = 6.6 assessments/day (SD = 3.5). *p‐value* < 10^–3^*.
							Median COMFORT‐B Scores: Pre‐implementation: median = 8 (range: 6–19) vs post‐implementation: median = 9.5 (range: 6–15.5). *p‐value*: 0.002*. Adequate Levels of Sedation (COMFORT‐B Scores 11–17): Pre‐implementation: 22.2% vs post‐implementation: 31.7%. *p‐value*: < 10^–3^*. Effect size: Increase of 9.5%. Excessive Sedation (COMFORT‐B Scores < 11): Pre‐implementation: 73.3% vs post‐implementation: 60.7%. *p‐value*: < 10^–3^*. Effect size: Reduction of 12.6%.
Neunhoeffer et al., 2015 [27]	Pre‐and‐post implementation study at a 14–bed medical‐surgical‐cardiac PICU.	Germany	337 non‐surgical patients, with PICU LOS > 24 hours.	pASP introduced to nursing staff in December 2011, with training and support provided. Decision trees for medication use. Nurses allowed to titrate medications based on scores to assess sedation and analgesia. Physicians could deviate from the protocol for patient safety. Structured approach to weaning sedatives and opioids, guided by the SOS to assess withdrawal symptoms.	Pain and sedation: COMFORT‐B and NISS.	SOS with a score of ≥ 4 indicating withdrawal symptoms.	**Total Dose of Benzodiazepines** Pre‐implementation: 5.9 mg/kg (range: 0–82.0) vs post‐implementation: 4.2 mg/kg (range: 0–66). *p‐value* = 0.009*. Effect size: Reduction of 1.7 mg/kg on average. **Total Dose of Opioids** Pre‐implementation: 3.9 mg/kg (range 0.1–70) vs post‐implementation: 3.1 mg/kg (range: 0.05–56). *p‐value* = 0.38. Effect size: Reduction of 0.8 mg/kg (non‐significant). **Rate of Withdrawal (SOS ≥ 4)** Pre‐implementation: 23.6% (39/165) vs post‐implementation: 12.8% (22/172). *p‐value* = 0.009*. Effect size: Reduction of 10.8%.
							**Duration of Mechanical Ventilation (Days)** Pre‐implementation: median = 2.02 days (range: 0.96–25.0) vs post‐implementation: median = 1.71 days (range: 0.96–66.0). *p‐value* = 0.23. Effect size: Reduction of 0.31 days (non–significant). **PICU Length of Stay (Days)** Pre‐implementation: median = 5.8 days (range: 1–37.75) vs post‐implementation: median = 5.0 days (range: 1–120). *p‐value* = 0.14. Effect size: Reduction of 0.8 days (non–significant).
Neunhoeffer et al., 2017 [28]	Pre‐and‐post implementation study at a 14–bed PICU.	Germany	226 patients, post‐op (general), 1–16 years old, receiving MV, and LOS > 24 hours.	pASP. Medications: Fentanyl: Continuous IV infusion (0.1–4 μg/kg/h, starting dose 0.5 μg/kg/h) for pain management. Midazolam: Continuous IV infusion (0.04–0.42 mg/kg/h, starting dose 0.04 mg/kg/h) for sedation. Clonidine: Continuous IV infusion (0.04–3 μg/kg/h, starting dose 0.04 μg/kg/h) after 48 hours. Melatonin: Oral (3–5 mg/day) after 3 days to regulate day‐night cycles.	Pain and sedation: COMFORT‐B and NISS.	SOS with a score of ≥ 4 indicating withdrawal symptoms.	**Benzodiazepine Exposure** Total dose (mg/kg): Pre‐implementation: median = 5.0 mg/kg (range: 0.5–58.0) vs post‐implementation: median = 4.0 mg/kg (range: 0.0–47). *p‐value* = 0.021*. Daily dose (mg/kg/day): Pre‐implementation: median = 4.4 mg/kg/day (range: 1.1–33.9) vs post‐implementation: median = 2.9 mg/kg/day (range: 0.0–9.9). *p‐value* < 0.001*. **Opioid Exposure** Total dose (mg/kg): Pre‐implementation: median = 5.0 mg/kg (range: 0.1–67.0) vs post‐implementation: median = 3.0 mg/kg (range: 0.1–71.0). *p‐value* = 0.81.
				Chloral hydrate: Oral (up to 25 mg/kg, four times daily) after second postoperative day to promote sleep. Propofol: Not used in patients < 16 years old.			Daily dose (mg/kg/day): Pre‐implementation: median = 0.7 mg/kg/day (range: 0.0–7.0) vs post‐implementation: median = 0.8 mg/kg/day (range: 0.0–3.7). *p‐value* = 0.35. **Rate of Withdrawal (SOS ≥ 4)** Pre‐implementation: 35.3% (41/116) vs post‐implementation: 20.0% (22/110). *p‐value* = 0.01*. **Duration of Mechanical Ventilation (Days)** Pre‐implementation: median = 0.9 days (range: 0.1–53.7) vs post‐implementation: median = 1.0 days (range: 0.3–28.0). *p‐value* = 0.81. **Length of PICU Stay (Days)** Pre‐implementation: median = 3.3 days (range: 1.0–53.8) vs post‐implementation: median = 3.0 days (range: 1.0–46.7). *p‐value* = 0.59.
Michel et al., 2020 [29]	Pre‐ and post‐modification study at a 14‐bed PICU. 2‐phase study consisting of a pre‐modification period using the original version of the analgesia and sedation protocol from January to December 2016 followed by a post‐modification period after modification of the protocol from January to December 2018. A period of 1 year was interposed between the two observation periods to ensure that the new protocol was implemented consistently.	Germany	65 patients, < 6 months old, post–cardiac surgery with CPB	Original Analgesia and Sedation Protocol (pre‐modification group): continuous IV infusions of morphine (30 mg/kg/h), midazolam (0.1 mg/kg/h), and clonidine (0.05 mg/kg/h). Clonidine was preferred over dexmedetomidine due to its availability in Europe, lower cost, and similar sedative effects. Sedation and analgesia levels were measured using the COMFORT‐B scale and the NISS. Medication was adjusted to achieve specific target ranges on the COMFORT‐B scale: 10–12 in the first 12 h and 12–18 thereafter. Boluses and increased doses of morphine and midazolam were given if required. The SOS‐PD scale monitored for withdrawal and delirium during weaning.	Pain and sedation: COMFORT‐B and NISS.	SOS–PD	**Cumulative Morphine (mg/kg)** Control: 332.7 (SD = 188.2) vs protocol: 367.8 (SD = 172.5). Difference: 35.1 mg/kg (95% CI: –124.6, 54.5). *p‐value*: 0.44. **Cumulative Midazolam (mg/kg)** Control: 1.17 (SD = 0.84) vs protocol: 0.67 (SD = 0.74). Difference: 0.50 mg/kg (95% CI: 0.11, 0.90). *p‐value*: 0.014*. **Patients with Withdrawal Symptoms** Control: 3 patients (9.1%) vs protocol: 5 patients (15.6%). *p‐value*: 0.48. **Duration of Mechanical Ventilation (Hours)** Control: 94.3 (SD = 80.3) hours vs protocol: 108.8 (SD = 85.33) hours. Difference: 14.5 h (95% CI: –55.58, 26.53). *p‐value*: 0.48. **PICU Length of Stay (Days)** Control: 9.7 (SD = 13.9) days vs protocol: 10.1 (SD = 5.8) days. Difference: 0.17 days (95% CI: –5.79, 4.86). *p‐value*: 0.86.
				Modified Analgesia and Sedation Protocol (post‐modification group): In the modified protocol, midazolam was not routinely used. Instead, sedation was primarily managed with morphine (30 mg/kg/h) and clonidine (0.2 mg/kg/h). Midazolam could be used at the discretion of the attending intensivist in case of under‐sedation, based on patient distress or agitation. The target sedation goals, and medication titration specifications (as well as weaning procedures) remained unchanged from the original protocol.			
Gaillard–Le Roux et al., 2017 [30]	A single‐centre prospective before and after study conducted at a 12‐bed surgical and medical PICU.	France	235 medical and surgical patients (194 included in analysis)	Nurse‐driven sedation protocol. Sedative administration: Protocol group: a structured sedation protocol guided sedative administration based on COMFORT‐B target ranges. Nurses adjusted doses every 10 min initially and assessed sedation every 3 h or more frequently if needed. Predefined maximum drug doses required physician approval for adjustment. Control group: sedation was managed at the physician's discretion. Nurses could not independently adjust sedative dosages.	Pain and sedation: COMFORT‐B	SOS	**Opioid Exposure** *Sufentanil* Daily dose: control group: median = 14.7 mcg/kg/day (IQR: 10.7–21.4) vs protocol group: median = 14.2 mcg/kg/day (IQR: 8.7–23.5). *p‐value* = 0.86. Length of infusion: control group: median = 3 days (IQR: 2–5) vs protocol group: median = 3 days (IQR: 2–5). *p‐value* = 0.58. *Morphine* Daily dose: control group: median = 0.34 mg/kg/day (IQR: 0.25–0.5) vs protocol group: median = 0.34 mg/kg/day (IQR: 0.25–0.45). *p‐value* = 0.73. Length of infusion: control group: median = 4 days (IQR: 3–7) vs protocol group: median = 5 days (IQR: 3–7). *p‐value* = 0.86. **Benzodiazepine Exposure** *Midazolam* Daily dose: control group: median = 1.2 mg/kg/day (IQR: 0.85–2.4) vs protocol group: median = 1.0 mg/kg/day (IQR: 0.56–1.8). *p‐value* = 0.02*. Length of infusion: control group: median = 4 days (IQR: 2–4) vs protocol group: median = 3 days (IQR: 2–6). *p‐value* = 0.26.
							**Incidence of Withdrawal Symptoms** No significant difference was highlighted between the two groups (missing data). **Duration of Mechanical Ventilation (Days)** Control group: 5 days (IQR: 3–7.5) vs protocol group: 4 days (IQR: 3–8). *p‐value* = 0.44. Subgroup analysis: patients older than 12 months Protocol group: 4 days (IQR: 3–8) vs control group: 5 days (IQR: 2.75–11.25). *p‐value* = 0.04*. Effect size: moderate, indicating a statistically significant shorter duration of MV in the protocol group compared to the control group for patients older than 12 months. **PICU Length of Stay (Days)** Control group: 7 days (IQR: 4–11.5) vs protocol group: 7 days (IQR: 5–12). *p‐value* = 0.42.
							**Documentation of Pain/Sedation Scores** Missing data on COMFORT‐B Scale: control group: 20% of patients had missing data on COMFORT‐B scale assessments vs protocol group: 4.1% of patients had missing data. *p‐value* = 0.004*. Effect size: large, suggesting a significant improvement in the completeness of COMFORT‐B scale documentation in the protocol group. COMFORT‐B Scale in control group: mean = 11.71 (SD = 2.7) vs protocol group: mean = 11.61 (SD = 1.9). *p‐value* = 0.93.
Larson and McKeever, 2018 [31]	Retrospective audit of patient medical records at a tertiary PICU pre and post introduction of an analgesic and sedative protocol.	Australia	100 post‐cardiac surgery patients	Protocol developed with contributions from nursing, medical and pharmacy teams and introduced following in‐service education for both nursing and medical staff. Escalation of pharmacological treatment: optimise post‐operative analgesia by providing boluses of morphine before increasing infusion rates.	Pain and sedation: COMFORT‐B	Not assessed	**Opioid Exposure** *Morphine* Children < 50 kg: pre‐protocol: mean = 23.86 (SD = 10) mcg/kg/h vs post‐protocol: mean = 18.37 (SD = 8.9) mcg/kg/h. *p‐value* = 0.006* (95% CI: 0.003–0.997). Children > 50 kg: pre‐protocol: median = 20 (IQR: 16–21) mcg/kg/h vs post‐protocol: median = 15 (IQR: 10–18) mcg/kg/h. *p‐value* = 0.12.
				Sedation protocol: clonidine recommended over midazolam as the first‐line sedative agent. Staff education: medical and nursing leads ensured ongoing staff education. Protocol availability: printed protocols available at each bedside. Online protocols accessible on the department intranet.			*Fentanyl* Data was not consistently reported across groups. **Benzodiazepine Exposure** *Midazolam* Children < 50 kg: pre‐protocol: median = 57.6 (IQR: 30.8–94.6) mcg/kg/h vs post‐protocol: median = 24.5 (IQR: 10–48.9) mcg/kg/h. *p‐value* = 0.024*. Children > 50 kg: pre‐protocol: median = 20.97 (IQR: 8.8–33) mcg/kg/h vs post‐protocol: median = 8.29 mcg/kg/h. *p‐value* = 0.22. **Median Duration of Ventilation (Hours)** Group 1: median = 11.53 h (IQR: 8.16–23.33) vs group 2: median = 18.95 h (IQR: 11.58–35.33). *p‐value* = 0.008* (statistically significant increase). **Median PICU Length of Stay (Hours)** Group 1: median = 27.5 h (IQR: 23.25–49.25) vs group 2: median = 47.33 h (IQR: 25.33–70.5). *p‐value* = 0.069. **Documentation of Pain/Sedation Scores** Patients assessed by COMFORT‐B pre‐protocol: 41 patients (82%) vs post‐protocol: 47 patients (94%). *p‐value* = 0.06.
							Frequency of assessment in 24 Hours: pre‐protocol: median = 3 assessments (IQR: 1–5) vs post‐protocol: median = 5 assessments (IQR: 3–7). *p‐value* = 0.006*. Score range < 10: pre‐protocol: 7 patients (14%) vs post‐protocol: 11 patients (22%). *p‐value* =0.29. Score Range 10–20: pre‐protocol: 5 patients (10%) vs post‐protocol: 12 patients (24%). *p‐value* = 0.062. Score Range > 20: pre‐protocol: 1 patient (2%) vs post‐protocol: 4 patients (8%). *p‐value* = 0.16.
Hanser et al., 2020 [32]	Retrospective pre‐ and post‐implementation study conducted in a cardiac PICU. Two cohorts of patients who underwent corrective surgery for cardiac TOF below the age of 7 months, were retrospectively evaluated before and after implementation of a nurse–driven analgesia and sedation protocol.	Germany	65 post–op patients for TOF	Nurse–driven analgesia and sedation protocol. Sedation and analgesia management: Continuous intravenous infusion of morphine and midazolam maintained as standard practice, with no change in initial dosages. Clonidine added after 24 h of PICU stay in the post‐implementation period. Adjuvant therapies (e.g., oral melatonin and chloral hydrate) consistent across pre‐ and post‐implementation periods. Nurse‐led sedative adjustments:	Pain and sedation: COMFORT‐B and NISS.	Not assessed with specific tool.	**Exposure to Opioids** *Morphine* Cumulative dose (mcg/kg): Pre‐implementation: 1,920 (IQR: 904–2,687) vs post‐implementation: 1,961 (IQR: 1,113–3,162). *p‐value* = 0.54. Peak dose (mcg/kg/hr): Pre‐implementation: 50.0 (IQR: 39.7–79.9) vs post‐implementation: 42.5 (IQR: 29.7–51.8). *p‐value*: 0.004*. **Exposure to Benzodiazepines** *Midazolam* Cumulative dose (mg/kg): Pre‐implementation: 7.37 (IQR: 4.70–17.65) vs post‐implementation: 5.0 (IQR: 2.70–9.12). *p‐value*: 0.014*. Peak dose (mg/kg/hr): Pre‐implementation: 0.22 (IQR: 0.20–0.33) vs post‐implementation: 0.15 (IQR: 0.13–0.20). *p‐value*: 0.001*.
				Nurses assessed sedation levels at least every 8 h using both the COMFORT‐B and NISS scales. In cases of discrepancy between COMFORT‐B and NISS, the NISS score took precedence. Infusions adjusted and boluses given according to pain and sedation scores. Medication tapering: Triggered by prolonged opioid/sedative use (> 3 days), readiness for extubation, or reduced analgesia requirements. Tapering Plan: Therapy < 5 days: reduce by 50% every 24 hours. Therapy > 5 days: reduce by 10–20% every 24 hours. Tapering was paused for 24 h if withdrawal symptoms occurred. Protocol deviations were allowed for patient safety as determined by the nursing staff or attending physicians.			**Duration of Mechanical Ventilation (Hours)** Pre‐implementation: 72 h (IQR: 24–141 h) vs post‐implementation: 49 h (IQR: 24–98 h). *p‐value*: 0.407. **PICU Length of Stay (Days)** Pre‐implementation: 7 days (IQR: 5–14 days) vs post‐implementation: 5 days (IQR: 4–7 days). *p‐value*: 0.017*.

Abbreviations: CI, confidence interval; CICU, cardiac intensive care unit; COMFORT‐B, COMFORT‐Behaviour scale; CPB, cardiopulmonary bypass; ERT, extubation readiness tests; FLACC, face, legs, activity, cry, consolability; IQR, interquartile range; IV, intravenous; LOS, length of stay; MV, mechanical ventilation; NISS, nurse interpretation of sedation scale; NRS, numerical rating scale; pASP, paediatric analgesia and sedation protocol; PICU, paediatric intensive care unit; RESTORE, randomised evaluation of sedation titration for respiratory failure; SBS, state behavioural scale; SOS, sophia observational withdrawal symptoms scale; SOS–PD, sophia observational withdrawal symptoms–paediatric delirium scale; TOF, tetralogy of fallot; WAT, withdrawal assessment tool; *, statistically significant.

### 5.3 Reduction in Opioid Doses

**FIGURE 2:**
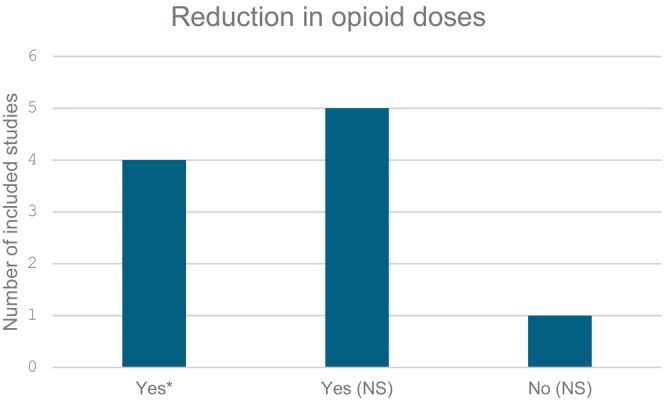
Reduction in opioid doses. * indicates statistically significant (*p* < 0.05). NS: statistically not significant.

Although the majority of studies included in this review reported no statistically significant variation in opioid dosing following the implementation of a nurse‐led analgesia and sedation protocol [24, 25, 26, 27, 28, 30], some studies demonstrated significant reductions [22, 23, 29, 31]. Notably, the cardiac‐RESTORE project demonstrated a statistically significant reduction in cumulative, average, and peak opioid doses for both morphine and fentanyl post‐implementation (morphine: cumulative dose (mg/kg/visit): median dose reduced from 1.12 (0.28–5.92) to 0.60 (0.25–2.23). *p* value = 0.008*. Morphine average dose (mg/kg/day): median dose reduced from 0.21 (0.10–0.62) to 0.15 (0.07–0.38). *p* value = 0.005*. Morphine peak dose (mg/kg/day): median dose reduced from 0.51 (0.15–2.15) to 0.29 (0.15–0.95). *p* value = 0.007*. Fentanyl cumulative dose (mcg/kg/visit): median dose reduced from 8.97 (1.40–36.6) to 2.73 (0.39–12.2). *p* value < 0.001*. Fentanyl average dose (mcg/kg/day): median dose reduced from 1.15 (0.46–3.03) to 0.93 (0.31–2.41). *p* value = 0.023*. Fentanyl peak dose (mcg/kg/day): median dose reduced from 2.95 (0.94–6.97) to 1.99 (0.39–5.16). *p* value = 0.002*) [23]. The original RESTORE trial reported fewer days of opioid exposure in patients receiving the intervention (9 days [IQR: 5–15] vs. control: 10 days [IQR: 4–20]. *p* value = 0.01*), although there was no significant difference in cumulative opioid doses between groups (cumulative dose (mg/kg): intervention: 13.7 (5.3–38.3) vs. control: 17.7 (5.3–52.9). *p* value = 0.10) [22]. Larson and McKeever (2018) observed a statistically significant reduction in morphine dosing rates among children weighing less than 50 kg (pre‐protocol: mean = 23.86 ± 10 mcg/kg/h vs. post‐protocol: mean = 18.37 ± 8.9 mcg/kg/h (95% CI: 0.003‐0.997). *p* value = 0.006*) [29]. Additionally, Hanser et al. (2020) found a significant decrease in peak morphine doses (peak dose (mcg/kg/h): pre‐implementation: 50.0 (IQR: 39.7–79.9) vs. post‐implementation: 42.5 (IQR: 29.7–51.8). *p* value: 0.004*), although cumulative morphine doses showed no significant reduction (cumulative dose (mcg/kg): pre‐implementation: 1920 (IQR: 904–2687) vs. post‐implementation: 1961 (IQR: 1113–3162). *p* value = 0.54) [31]. These findings are summarised and illustrated in Figure 2.

### 5.4 Reduction in Benzodiazepine Doses

**FIGURE 3:**
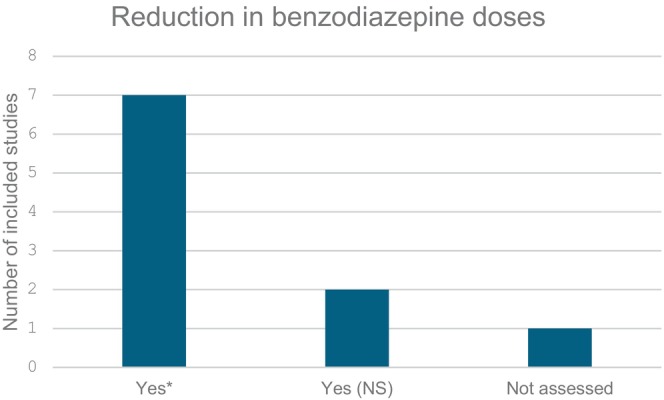
Reduction in benzodiazepine doses. * indicates statistically significant (*p* < 0.05). NS: statistically not significant.

The majority of included studies reported a statistically significant reduction in benzodiazepine doses administered following the implementation of a nurse‐led analgesia and sedation protocol [23, 24, 27, 28, 29, 30, 31]. Notably, reductions in cumulative doses were demonstrated by half of the studies. For example, Lincoln et al. (2020) observed a significant decrease in midazolam cumulative doses (midazolam: cumulative dose (mg/kg/visit): median dose reduced from 0.60 (0.12–3.68) to 0.25 (0.08–1.09) post‐implementation. *p* value < 0.001*) during the cardiac‐RESTORE project [23]. Similarly, Neunhoeffer et al. (2015) found a reduction in total benzodiazepine doses (benzodiazepine total dose (mg/kg): pre‐implementation: 5.9 (range: 0–82.0) vs. post‐implementation: 4.2 (range: 0–66). *p* value = 0.009*) [27]. Further reductions were reported by Neunhoeffer et al. (2017) (benzodiazepine total dose (mg/kg): pre‐implementation: median = 5.0 (range: 0.5–58.0) vs. post‐implementation: median = 4.0 (range: 0.0–47). *p* value = 0.021*) [28], Michel et al. (2020) (cumulative midazolam (mg/kg) control: 1.17 ± 0.84 vs. protocol: 0.67 ± 0.74. Difference: 0.50 mg/kg (95% CI: 0.11, 0.90). *p* value: 0.014*) [24], and Hanser et al. (2020) (midazolam cumulative dose (mg/kg): pre‐implementation: 7.37 (IQR: 4.70–17.65) vs. post‐implementation: 5.0 (IQR: 2.70–9.12). *p* value: 0.014*) [31]. In addition, significant decreases in daily benzodiazepine doses were reported. Gaillard‐Le Roux et al. (2017) observed a reduction in the median daily midazolam dose (midazolam daily dose (mg/kg/day): control group: median = 1.2 (IQR: 0.85–2.4) vs. protocol group: median = 1.0 (IQR: 0.56–1.8). *p* value = 0.02*) [30]. Larson and McKeever (2018) demonstrated a significant decrease in midazolam exposure among children weighing less than 50 kg (pre‐protocol: median = 57.6 (IQR: 30.8–94.6) mcg/kg/h vs. post‐protocol: median = 24.5 (IQR: 10–48.9) mcg/kg/h. *p* value = 0.024*) [29]. These findings are summarised and illustrated in Figure 3.

### 5.5 Documentation of Pain and Sedation Scores

Several studies in this review evaluated the frequency of assessment and the pain and sedation scores recorded by nurses before and after protocol implementation. For instance, Dreyfus et al. (2017) reported significant improvements associated with protocol use, including an increase in the frequency of COMFORT‐B assessments, a higher proportion of adequate sedation levels, and a reduction in oversedation. Specifically, the frequency of COMFORT‐B assessments increased from a mean of 3.9 assessments/day (SD = 2.5) pre‐implementation to 6.6 assessments/day (SD = 3.5) post‐implementation (*p* < 10^−3^). Adequate sedation levels (COMFORT‐B Scores 11–17) rose from 22.2% to 31.7% (*p* < 10^−3^; effect size: 9.5% increase), while excessive sedation (COMFORT‐B Scores < 11) decreased from 73.3% to 60.7% (*p* < 10^−3^; effect size: 12.6% reduction) [26]. Similarly, Gaillard‐Le Roux et al. (2017) observed a significant improvement in the completeness of COMFORT‐B scale documentation. Missing data on the COMFORT‐B scale occurred in 20% of patients in the control group compared to only 4.1% in the protocol group (*p* = 0.004*) [30]. Last, Larson and McKeever (2018) demonstrated a significant increase in the frequency of pain and sedation assessments using the COMFORT‐B scale, with the median number of assessments in 24 h rising from 3 (IQR: 1–5) pre‐protocol to 5 (IQR: 3–7) post‐protocol (*p* = 0.006*) [29].

### 5.6 Incidence of Iatrogenic Withdrawal Syndrome

**FIGURE 4:**
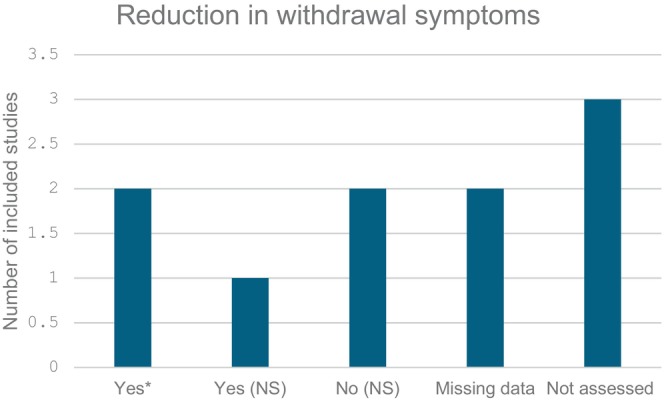
Reduction in withdrawal symptoms. * indicates statistically significant (*p* < 0.05). NS: statistically not significant.

Two German studies (Neunhoeffer et al. 2015; Neunhoeffer et al. 2017) implemented a standardised nurse‐driven paediatric analgesia and sedation protocol (pASP) for mechanically ventilated patients, utilising the Sophia Observation Withdrawal Symptoms scale (SOS). Both studies demonstrated a statistically significant reduction in withdrawal symptoms following protocol implementation. In the 2015 study, the incidence of withdrawal (SOS ≥ 4) decreased from 23.6% (39/165) pre‐implementation to 12.8% (22/172) post‐implementation (*p* = 0.009*; effect size: 10.8% reduction) [27]. Similarly, the 2017 study reported a reduction from 35.3% (41/116) to 20.0% (22/110) (*p* = 0.01*; effect size: 15.3% reduction) [28]. Additionally, both studies found a significant decrease in cumulative benzodiazepine exposure post‐protocol. In the 2015 study, the total dose of benzodiazepines was reduced from 5.9 mg/kg (range: 0–82.0) to 4.2 mg/kg (range: 0–66.0) (*p* = 0.009*) [27]. The 2017 study reported a reduction in total benzodiazepine dose from 5.0 mg/kg (range: 0.5–58.0) to 4.0 mg/kg (range: 0.0–47.0) (*p* = 0.021*), along with a decrease in daily benzodiazepine dose from 4.4 mg/kg/day (range: 1.1–33.9) to 2.9 mg/kg/day (range: 0.0–9.9) (*p* < 0.001*) [28]. In contrast, opioid exposure showed a non‐significant reduction in both studies. Conversely, two other studies (Dreyfus et al. 2017; Lincoln et al. 2020) reported a non‐significant reduction in IWS post‐implementation. Dreyfus et al. (2017) observed a decrease in the incidence of withdrawal symptoms from 23% (24/104) pre‐protocol to 14% (13/93) post‐protocol (*p* = 0.103), suggesting a possible trend toward improvement but without statistical significance [26]. Similarly, Lincoln et al. (2020) did not report a significant change in withdrawal incidence despite an overall reduction in sedative and opioid exposure [23]. On the other hand, Michel et al. (2020) observed a slight increase in IWS incidence after protocol modifications, rising from 9.1% (3/33) to 15.6% (5/32), though this difference was not statistically significant (*p* = 0.48) [24]. These findings highlight the variability in IWS outcomes across different protocols and settings, as summarised in Figure 4.

### 5.7 Duration of Mechanical Ventilation

Most studies assessing the impact of protocol implementation on the duration of mechanical ventilation (MV) did not demonstrate statistically significant changes. The cluster‐randomised controlled trial by Curley et al. (2015), which designated MV duration as the primary outcome, found no significant difference between the intervention and control groups (median 6.5 days [IQR: 4.1–11.2] vs. 6.5 days [IQR: 3.7–12.1], *p* = 0.61) [22]. However, two prospective before‐and‐after studies identified statistically significant reductions in MV duration in subgroup analyses. Lincoln et al. (2020) reported a substantial reduction in MV duration among patients categorised in lower Society of Thoracic Surgeons‐European Association for Cardio‐Thoracic Surgery (STAT) risk categories, reflecting lower mortality risk associated with cardiac surgical procedures (*p* < 0.001*) [23]. Likewise, Gaillard‐Le Roux et al. (2017) observed a significant reduction in MV duration among children older than 12 months, decreasing from a median of 5 days (IQR: 2.75–11.25) in the control group to 4 days (IQR: 3–8) post‐implementation (*p* = 0.04*) [30]. Conversely, a retrospective before‐and‐after study by Larson and McKeever (2018) found a statistically significant increase in MV duration following protocol implementation, with median MV duration rising from 11.53 h (IQR: 8.16–23.33) pre‐protocol to 18.95 h (IQR: 11.58–35.33) post‐protocol (*p* = 0.008*) [29].

### 5.8 PICU and Hospital Length of Stay

Most studies in this review found no statistically significant difference in PICU or hospital length of stay (LOS) following protocol implementation. The RCT by Curley et al. (2015) reported a median PICU stay of 9.6 days (IQR: 6.2–15.6) in the intervention group version. 9.6 days (IQR: 5.7–16.6) in the control group (*p* = 0.61), demonstrating no significant impact of the protocol [22]. Similarly, Dreyfus et al. (2017), Neunhoeffer et al. (2015, 2017), Michel et al. (2020) and Gaillard‐Le Roux et al. (2017) found non‐significant changes in PICU LOS, with *p* values ranging from 0.14 to 0.86 [24, 26, 27, 28, 30]. However, two studies focusing on post‐cardiac surgery patients identified statistically significant reductions in PICU and/or hospital LOS following the adoption of a nurse‐driven analgesia and sedation protocol. Hanser et al. (2020) reported a decrease in PICU LOS from a median of 7 days (IQR: 5–14) pre‐protocol to 5 days (IQR: 4–7) post‐protocol (*p* = 0.017*) in post‐cardiac surgery patients for Tetralogy of Fallot [31]. Similarly, Lincoln et al. (2020) found a significant reduction in hospital LOS among cardiac patients with lower STAT risk categories after protocol implementation (*p* < 0.05*) [23]. Additionally, these studies linked protocol implementation to significantly lower cumulative benzodiazepine doses and reductions in either cumulative or peak opioid doses during the post‐implementation period. In Hanser et al. (2020), cumulative midazolam exposure decreased from 7.37 mg/kg (IQR: 4.70–17.65) to 5.0 mg/kg (IQR: 2.70–9.12) (*p* = 0.014*), while peak midazolam dose was reduced from 0.22 mg/kg/h (IQR: 0.20–0.33) to 0.15 mg/kg/h (IQR: 0.13–0.20) (*p* = 0.001*) [31]. Similarly, Lincoln et al. (2020) found a significant reduction in morphine cumulative dose (*p* = 0.008*) and peak dose (*p* = 0.007*) post‐implementation [23].

## 6 Discussion

This systematic review found that nurse‐driven analgesia and sedation protocols significantly reduced benzodiazepine doses in critically ill children, with most studies reporting a statistically significant decrease in drug doses post‐implementation. These results align with both paediatric and adult literature, including a systematic review and meta‐analysis that demonstrated lower midazolam doses and reduced delirium in mechanically ventilated adults under nurse‐led sedation protocols [33]. Such protocols may also help decrease withdrawal symptoms in PICU patients, as suggested by Neunhoeffer et al. (2015; 2017), who found that higher benzodiazepine doses correlate with increased withdrawal symptoms, especially in post‐operative surgical patients [27, 28]. Additionally, benzodiazepine exposure, particularly peak doses of midazolam, has been linked to withdrawal and delirium in critically ill children [34, 35, 36]. However, Michel et al. (2020) observed a non‐significant increase in withdrawal symptoms following protocol modifications, potentially due to improved detection from recent staff training [24].

Given the positive association between benzodiazepines and the incidence of withdrawal and delirium, recent studies have been studying alternatives to benzodiazepines that can be used in clinical practice. Shildt et al. (2021) implemented a benzodiazepine‐sparing protocol in PICU [37], while Barends et al. (2017) found dexmedetomidine, a selective alpha‐2 adrenergic receptor agonist, to be a viable alternative to midazolam for procedural sedation [38]. Clonidine and dexmedetomidine are considered preferable to benzodiazepines due to their lower neurotoxicity, with dexmedetomidine offering advantages such as rapid onset, sedation that mimics natural sleep, reduced anxiety and minimal impact on respiratory function [39, 40]. Alpha‐2 agonists are recommended as primary sedatives for critically ill paediatric postoperative cardiac patients, especially when early extubation is anticipated [7]. This recommendation is supported by Hanser et al. (2020), who demonstrated that their sedation protocol led to a decreased PICU length of stay for patients undergoing cardiac surgery for Tetralogy of Fallot by altering the clonidine administration policy with the introduction of the protocol [31]. Before the protocol, clonidine was given several days post‐operation to aid in tapering analgesia and sedation. After the protocol's implementation, clonidine was administered as a continuous infusion starting 24 h after surgery. Similarly, Lincoln et al. (2020) found that the cardiac‐RESTORE protocol significantly reduced both PICU and hospital length of stay for post‐cardiac surgery patients, further supporting the benefits of structured sedation and analgesia protocols in this patient sub‐group [23].

This systematic review suggests that nurse‐led analgesia and sedation protocols are associated with a significant reduction in benzodiazepine doses, but they do not lead to the same degree of reduction in opioid doses. Since this conclusion is based on narrative synthesis, caution is needed in interpreting these findings as definitive. This discrepancy may be attributed to the complexities inherent in pain assessment within the PICU, where the diverse developmental stages of patients and conditions complicate the evaluation of pain [41]. Nurses face particular challenges in assessing pain in preverbal children or those with cognitive impairments [42]. Despite the statistically non‐significant reduction in opioid use following protocol implementation, these protocols have been shown to improve pain score documentation and may enhance nurses' confidence and assessment skills, suggesting potential benefits in patient care [26, 29, 30].

The results of this systematic review as well as the majority of paediatric research published to date have not demonstrated a clear association between protocol use and reduced MV duration [43, 44]. Protocols are theoretically expected to reduce MV duration by lowering doses of analgesics and sedatives that depress spontaneous ventilation, and several RCTs in adults have demonstrated improved outcomes, including reduced MV length, with protocol use [45]. However, similar results have not been confirmed in paediatric populations, possibly due to confounding factors such as hemodynamic instability and reintubation challenges [46, 47]. Larson and McKeever (2018) reported a significant increase in MV duration post‐protocol implementation, attributed to a baseline imbalance with younger patients in the post‐implementation group [29]. Accordingly, the study by Gaillard‐Le Roux et al. (2017) showed a significant reduction in MV duration in patients older than 12 months after implementing a nurse‐led sedation protocol [30]. Lincoln et al. (2020) also found a significant reduction in MV duration in subgroup analyses, particularly among patients with a lower mortality risk associated with cardiac surgical procedures [23]. These findings suggest that protocol implementation could shorten MV duration in older children and those at lower risk for postoperative complications.

## 7 Limitations

This systematic review adheres to a rigorous methodology, following PRISMA guidelines and the Cochrane Handbook while utilising validated tools throughout the assessment process. However, several limitations must be acknowledged. The search was restricted to four specialised databases, which, while relevant, may have led to publication bias and the omission of pertinent studies, affecting the generalizability of findings. A major limitation is the scarcity of RCTs, with most included studies relying on single‐arm, before‐and‐after designs that inherently carry a high risk of bias. The Cochrane risk‐of‐bias assessments revealed that the majority of studies exhibited serious risks, particularly due to confounding, selection bias, and selective reporting. These methodological weaknesses, combined with small sample sizes and retrospective designs, undermine the validity of the results. Furthermore, substantial heterogeneity in study designs, methods, and outcomes precluded meta‐analysis, necessitating a narrative synthesis that may be influenced by researcher interpretation. Another challenge lies in the variability of nurse‐led analgesia and sedation protocols across studies, including differences in sedation goals, tapering strategies, and levels of nurse autonomy, which were not systematically explored but could have significantly influenced outcomes. Despite these limitations, this review provides valuable insights into a complex clinical area, highlighting the need for more robust, well‐controlled studies to better inform practice in paediatric critical care.

## 8 Implications and Recommendations for Practice

The literature reviewed indicates that nurse‐led analgesia and sedation protocols in the PICU are both feasible and safe, particularly for specific patient subgroups. In paediatric cardiac surgery patients, for instance, protocolised sedation weaning may shorten both PICU and hospital stays, leading to improved patient outcomes and reduced healthcare costs. Further research should focus on these populations to generate high‐quality evidence that supports broader clinical integration of such protocols. However, challenges including patient heterogeneity, cultural barriers, limited evidence of direct patient benefit and the absence of clear implementation strategies may hinder their widespread use. Successful application may be more achievable in well‐staffed settings with structured training, robust auditing systems and ongoing professional education. The evolving role of critical care nurses in sedation management is integral to optimising patient outcomes, particularly in sedation weaning, where their assessments and clinical judgement are crucial in determining the timing and approach to reducing sedative medications. This increased autonomy necessitates comprehensive training to ensure consistent protocol adherence and enhance decision‐making capabilities. Beyond technical competence, training should emphasise critical thinking and clinical reasoning. Additionally, regular audits and continuous education are vital for maintaining protocol fidelity, identifying areas for refinement, and equipping nurses to tailor sedation practices to individual patient needs. Collectively, these measures could strengthen the quality and safety of sedation management in paediatric critical care.

## 9 Conclusion

Nurse‐driven analgesia and sedation protocols appear to be effective in reducing benzodiazepine exposure in critically ill children, particularly when combined with alpha‐2 adrenergic agents like clonidine. While a reduction in opioid use was observed, this finding was not statistically significant. Given the narrative synthesis approach, these conclusions should be interpreted with caution, and further high‐quality research is needed to more definitively assess the impact of these protocols on medication use and overall patient outcomes. The observed reduction in benzodiazepine use may be linked to a decreased incidence of iatrogenic withdrawal symptoms. Additionally, protocols may enhance nurses' confidence and competence in managing pain and sedation, fostering a more standardised approach within multidisciplinary teams. This systematic review also suggests a potential reduction in mechanical ventilation duration among older children and those with lower risk associated with cardiac surgery, as well as decreased PICU and hospital length of stay in post‐operative cardiac patients. Future research should concentrate on these specific patient groups to further validate these findings.

## Conflicts of Interest

The authors declare no conflicts of interest.

## Supporting information


**Figure S1.** Detailed search strategy used in the systematic review.

**Document 1.** Risk of bias assessment results for the RESTORE trial by Curley et al (2015).
**Document 2**. Risk of bias assessment results for the cardiac‐RESTORE by Lincoln et al (2020).
**Document 3**. Risk of bias assessment results for Magner et al (2020).
**Document 4**. Risk of bias assessment results for Dreyfus et al (2017).
**Document 5**. Risk of bias assessment results for Neunhoeffer et al (2015).
**Document 6**. Risk of bias assessment results for Neunhoeffer et al (2017).
**Document 7**. Risk of bias assessment results for Michel et al (2020).
**Document 8**. Risk of bias assessment results for Gaillard‐Le Roux et al (2017).
**Document 9**. Risk of bias assessment results for Larson and McKeever (2018).
**Document 10**. Risk of bias assessment results for Hanser et al (2020).

## Data Availability

All data generated or analysed during this study are included in the published manuscript and Supporting Information.
