# Supplementary material for: Impact of Nurse‐Driven Analgesia and Sedation Protocols on Medication Exposure and Withdrawal in Critically Ill Children: A Systematic Review
Source: Nurs Crit Care. 2025 May 29;30(3):e70051. doi: 10.1111/nicc.70051 (PMC12120589; doi:10.1111/nicc.70051)
Supplement: Supplementary file 1 — Figure S1. Detailed search strategy used in the systematic review. [file NICC-30-0-s001.pdf]

("nurse-driven" OR "nurse-directed" OR "nurse-led" OR "nurse-implemented") AND
(analgesia OR "pain relief" OR "pain management" OR sedation) AND
(opioids OR morphine OR benzodiazepines OR midazolam) AND
("paediatric intensive care unit" OR "pediatric intensive care unit" OR PICU OR "paediatric critical care unit" OR "pediatric critical care unit" OR "intensive care") AND
("critically ill paediatric patients" OR "critically ill pediatric patients" OR "children in PICU" OR "paediatric critical care" OR child OR pediatric OR paediatric)
