# Supplementary material for: Impact of Nurse‐Driven Analgesia and Sedation Protocols on Medication Exposure and Withdrawal in Critically Ill Children: A Systematic Review
Source: Nurs Crit Care. 2025 May 29;30(3):e70051. doi: 10.1111/nicc.70051 (PMC12120589; doi:10.1111/nicc.70051)
Supplement: Supplementary file 2 — Document 1. Risk of bias assessment results for the RESTORE trial by Curley et al (2015). Document 2. Risk of bias assessment results for the cardiac‐RESTORE by Lincoln et al (2020). Document 3. Risk of bias assessment results for Magner et al (2020). Document 4. Risk of bias assessment results for Dreyfus et al (2017). Document 5. Risk of bias assessment results for Neunhoeffer et al (2015). Document 6. Risk of bias assessment results for Neunhoeffer et al (2017). Document 7. Risk of bias assessment results for Michel et al (2020). Document 8. Risk of bias assessment results for Gaillard‐Le Roux et al (2017). Document 9. Risk of bias assessment results for Larson and McKeever (2018). Document 10. Risk of bias assessment results for Hanser et al (2020). [file NICC-30-0-s002.zip › Supplementary document 1.docx]

Revised Cochrane risk-of-bias tool for cluster-randomized trials (RoB 2 CRT)

TEMPLATE FOR COMPLETION

**Version of 18 March 2021**

The development of the RoB 2 tool was supported by the MRC Network of Hubs for Trials Methodology Research (MR/L004933/2- N61), with the support of the host MRC ConDuCT-II Hub (Collaboration and innovation for Difficult and Complex randomised controlled Trials In Invasive procedures - MR/K025643/1), by MRC research grant MR/M025209/1, and by a grant from The Cochrane Collaboration.


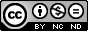


This work is licensed under a [Creative Commons Attribution-NonCommercial-NoDerivatives 4.0 International License](http://creativecommons.org/licenses/by-nc-nd/4.0/).

| **Study details**   \| **Reference** \| Curley, M. A. Q., Wypij, D., Watson, R. S., Grant, M. J. C., Asaro, L. A., Cheifetz, I. M., Dodson, B. L., Franck, L. S., Gedeit, R. G., Angus, D. C. and Matthay, M. A. (2015) Protocolized sedation vs usual care in pediatric patients mechanically ventilated for acute respiratory failure: A randomized clinical trial, JAMA : The Journal of the American Medical Association, 313 (4), pp. 379-389. DOI: 10.1001/jama.2014.18399. \| \| --- \| --- \|   **Study design**   \| □ \| Individually-randomized parallel-group trial \| \| --- \| --- \| \| X \| Cluster-randomized parallel-group trial \| \| □ \| Individually randomized cross-over (or other matched) trial \|   **For the purposes of this assessment, the interventions being compared are defined as**   \| Experimental: \| RESTORE protocol \| Comparator: \| Usual care \| \| --- \| --- \| --- \| --- \|  \| **Specify which outcome is being assessed for risk of bias** \|  \| \| --- \| --- \|  \| **Specify the numerical result being assessed.** In case of multiple alternative analyses being presented, specify the numeric result (e.g. RR = 1.52 (95% CI 0.83 to 2.77) and/or a reference (e.g. to a table, figure or paragraph) that uniquely defines the result being assessed. \|  \| \| --- \| --- \|   **Is the review team’s aim for this result…?**   \| □ \| to assess the effect of *assignment to intervention* (the ‘intention-to-treat’ effect) \| \| --- \| --- \| \| □ \| to assess the effect of *adhering to intervention* (the ‘per-protocol’ effect) \|   **If the aim is to assess the effect of *adhering to intervention***, select the deviations from intended intervention that should be addressed (at least one must be checked):  □ occurrence of non-protocol interventions  □ failures in implementing the intervention that could have affected the outcome  □ non-adherence to their assigned intervention by trial participants  **Which of the following sources were obtained to help inform the risk-of-bias assessment? (tick as many as apply)**  □ Journal article(s) with results of the trial  □ Trial protocol  □ Statistical analysis plan (SAP)  □ Non-commercial trial registry record (e.g. ClinicalTrials.gov record)  □ Company-owned trial registry record (e.g. GSK Clinical Study Register record)  □ “Grey literature” (e.g. unpublished thesis)  □ Conference abstract(s) about the trial  □ Regulatory document (e.g. Clinical Study Report, Drug Approval Package)  □ Research ethics application  □ Grant database summary (e.g. NIH RePORTER or Research Councils UK Gateway to Research)  □ Personal communication with trialist  □ Personal communication with the sponsor |
| --- | --- | --- | --- | --- | --- | --- | --- | --- | --- | --- | --- | --- | --- | --- | --- | --- | --- | --- | --- | --- |

## Risk of bias assessment

Responses underlined in green are potential markers for low risk of bias, and responses in red are potential markers for a risk of bias. Where questions relate only to sign posts to other questions, no formatting is used.

**Domain 1a: Risk of bias arising from the randomization process**

| **Signalling questions** | **Comments** | **Response options** |
| --- | --- | --- |
| **1a.1 Was the allocation sequence random?** | **1a.1** YES. The use of a computer-generated random sequence, stratified by PICU size, indicates a robust randomization process. **Judgment:** Y  **1a.2** The study does not specifically describe allocation concealment, but it is implied by the cluster-randomization design and use of computer-generated sequences. **Judgment:** PY | Y / PY / PN / N / NI |
| **1a.2 Was the allocation sequence concealed until clusters were enrolled and assigned to interventions?** |  | Y / PY / PN / N / NI |
| **1a.3 Did baseline differences between intervention groups suggest a problem with the randomization process?** | **1a.3** The groups were largely balanced at baseline, except for slightly younger patients and more cases of bronchiolitis in the intervention group. While these differences exist, they are minor and unlikely to indicate an issue with randomization. **Judgment:** N | Y / PY / PN / N / NI |
| **Risk-of-bias judgement** | **Low risk** | Low / High / Some concerns |
| Optional: What is the predicted direction of bias arising from the randomization process? |  | NA / Favours experimental / Favours comparator / Towards null /Away from null / Unpredictable |

**Domain 1b: Risk of bias arising from the timing of identification or recruitment of participants in a cluster-randomized trial**

| **Signalling questions** | **Comments** | **Response options** |
| --- | --- | --- |
| **1b.1 Were all the individual participants identified and recruited (if appropriate) before randomization of clusters?** | **1b.1** NO. In cluster-randomized trials, participants are often recruited after randomization. In this study, participants were enrolled after their PICU had already been assigned to an intervention. **Judgment:** N | Y/PY/PN/N/NI |
| **1b.2 If N/PN/NI to 1b.1: Is it likely that selection of individual participants was affected by knowledge of the intervention assigned to the cluster?** | **1b.2** YES. The consent rates were lower in intervention PICUs (72% vs. 84%, p=0.01), suggesting knowledge of the intervention may have influenced recruitment or consent decisions. **Judgment:** Y | NA/Y/PY/PN/N/NI |
| **1b.3 Were there baseline imbalances that suggest differential identification or recruitment of individual participants between intervention groups?** | **1b.3** YES. Intervention sites enrolled slightly more patients under 2 years of age and more cases of bronchiolitis. These imbalances may have arisen due to differential recruitment strategies. **Judgment:** Y | Y/PY/PN/N/NI |
| **Risk-of-bias judgement** | **Some concerns (due to recruitment bias)** | Low / High / Some concerns |
| Optional: What is the predicted direction of bias arising from the timing of identification and recruitment of participants? |  | NA / Favours experimental / Favours comparator / Towards null /Away from null / Unpredictable |

Domain 2: Risk of bias due to deviations from the intended interventions (*effect of assignment to intervention*)

| **Signalling questions** | **Comments** | **Response options** |
| --- | --- | --- |
| **2.1a Were participants aware that they were in a trial?** | **2.1a** YES. Participants were likely informed, as is standard in clinical trials for ethical reasons. **Judgment:** Y | Y / PY / PN / N / NI |
| **2.1b. If Y/PY/NI to 2.1a: Were participants aware of their assigned intervention during the trial?** | **2.1b** YES. Participants and caregivers would have been aware of the intervention (protocolized sedation vs. usual care) due to its observable nature. **Judgment:** Y  **2.2** YES. The care providers were necessarily aware, as they were responsible for implementing the intervention. **Judgment:** Y | NA / Y / PY / PN / N / NI |
| **2.2. Were carers and people delivering the interventions aware of participants' assigned intervention during the trial?** |  | Y / PY / PN / N / NI |
| **2.3. If Y/PY/NI to 2.1 or 2.2: Were there deviations from the intended intervention that arose because of the trial context?** | **2.3** NO. Protocol fidelity was high in the intervention group (94% adherence at enrollment), and there is no evidence of systematic deviations. **Judgment:** N | NA / Y / PY / PN / N / NI |
| **2.4 If Y/PY to 2.3: Were these deviations likely to have affected the outcome?** | **2.4** Not applicable, as there were no significant deviations from the protocol. **Judgment:** NA | NA / Y / PY / PN / N / NI |
| **2.5. If Y/PY/NI to 2.4: Were these deviations from intended intervention balanced between groups?** | **2.5** NA  **Judgment:** NA | NA / Y / PY / PN / N / NI |
| **2.6 Was an appropriate analysis used to estimate the effect of assignment to intervention?** | **2.6** YES. The study used an intention-to-treat (ITT) analysis, adjusting for key baseline covariates like age and PRISM III-12 scores. **Judgment:** Y | Y / PY / PN / N / NI |
| **2.7 If N/PN/NI to 2.6: Was there potential for a substantial impact (on the result) of the failure to analyse participants in the group to which they were randomized?** | **2.7** Not applicable. ITT analysis was conducted. **Judgment:** NA | NA / Y / PY / PN / N / NI |
| **Risk-of-bias judgement** | **Low risk** | Low / High / Some concerns |
| Optional: What is the predicted direction of bias due to deviations from intended interventions? |  | NA / Favours experimental / Favours comparator / Towards null /Away from null / Unpredictable |

Domain 2: Risk of bias due to deviations from the intended interventions (*effect of adhering to intervention*)

| **Signalling questions** | **Comments** | **Response options** |
| --- | --- | --- |
| **2.1. Were participants aware of their assigned intervention during the trial?** |  | Y / PY / PN / N / NI |
| **2.2. Were carers and people delivering the interventions aware of participants' assigned intervention during the trial?** |  | Y / PY / PN / N / NI |
| **2.3. [If applicable:] If Y/PY/NI to 2.1 or 2.2: Were important non-protocol interventions balanced across intervention groups?** |  | NA / Y / PY / PN / N / NI |
| **2.4. [If applicable:] Were there failures in implementing the intervention that could have affected the outcome?** |  | NA / Y / PY / PN / N / NI |
| **2.5. [If applicable:] Was there non-adherence to the assigned intervention regimen that could have affected participants’ outcomes?** |  | NA / Y / PY / PN / N / NI |
| **2.6. If N/PN/NI to 2.3, or Y/PY/NI to 2.4 or 2.5: Was an appropriate analysis used to estimate the effect of adhering to the intervention?** |  | NA / Y / PY / PN / N / NI |
| **Risk-of-bias judgement** |  | Low / High / Some concerns |
| Optional: What is the predicted direction of bias due to deviations from intended interventions? |  | NA / Favours experimental / Favours comparator / Towards null /Away from null / Unpredictable |

Domain 3: Risk of bias due to missing outcome data

| **Signalling questions** | **Comments** | **Response options** |
| --- | --- | --- |
| **3.1a Were data for this outcome available for all clusters that recruited participants?** | **3.1a** YES. Data were included for all patients except those who withdrew consent (n=10). Data from 15 patients who withdrew from the intervention but allowed data collection were included. **Judgment:** Y | Y / PY / PN / N / NI |
| **3.1b Were data for this outcome available for all, or nearly all, participants within clusters?** | **3.1b** Data were largely available, but there were some exclusions due to withdrawals and other factors (e.g., 25 patients withdrawn in intervention group, with partial data retained for 15 patients).  **Judgment:** **PY (Probably Yes)** | Y / PY / PN / N / NI |
| **3.2 If N/PN/NI to 3.1a or 3.1b: Is there evidence that the result was not biased by missing data?** | **3.2** Data were mostly complete, so this question is not applicable.  **Judgment:** NA | NA / Y / PY / PN / N |
| **3.3 If N/PN to 3.2 Could missingness in the outcome depend on its true value?** | **3.3** NA  **Judgment:** NA  **3.4** NA  **Judgment:** NA | NA / Y / PY / PN / N / NI |
| **3.4 If Y/PY/NI to 3.3: Is it likely that missingness in the outcome depended on its true value?** |  | NA / Y / PY / PN / N / NI |
| **Risk-of-bias judgement** | **Low risk** | Low / High / Some concerns |
| Optional: What is the predicted direction of bias due to missing outcome data? |  | NA / Favours experimental / Favours comparator / Towards null /Away from null / Unpredictable |

Domain 4: Risk of bias in measurement of the outcome

| **Signalling questions** | **Comments** | **Response options** |
| --- | --- | --- |
| **4.1 Was the method of measuring the outcome inappropriate?** | **4.1** The outcome measures were appropriate, as reported methods adhered to standard practices.  **Judgment:** N | Y / PY / PN / N / NI |
| **4.2 Could measurement or ascertainment of the outcome have differed between intervention groups?** | **4.2** Variability in adherence to protocol assessments (e.g., sedation and withdrawal evaluations) between groups may have introduced differences.  **Judgment:** PY | Y / PY / PN / N / NI |
| **4.3a If N/PN/NI to 4.1 and 4.2: Were outcome assessors aware that a trial was taking place?** | **4.3a** Not applicable, as some bias due to differences in outcome assessment between groups was indicated.  **Judgment:** NA | NA / Y / PY / PN / N / NI |
| **4.3b If Y/PY/NI to 4.3a: Were outcome assessors aware of the intervention received by study participants?** | **4.3b** Likely, as intervention strategies were explicit.  **Judgment:** PY | NA / Y / PY / PN / N / NI |
| **4.4 If Y/PY/NI to 4.3b: Could assessment of the outcome have been influenced by knowledge of intervention received?** | **4.4** Knowledge of the intervention might have influenced assessments such as sedation-related outcomes.  **Judgment:** PY  **4.5** Any influence appears limited and not systematic.  **Judgment:** PN | NA / Y / PY / PN / N / NI |
| **4.5 If Y/PY/NI to 4.4: Is it likely that assessment of the outcome was influenced by knowledge of intervention received?** |  | NA / Y / PY / PN / N / NI |
| **Risk-of-bias judgement** | **Some concerns** Comment: Awareness of intervention group assignments introduces potential bias, though likely minimal. | Low / High / Some concerns |
| Optional: What is the predicted direction of bias in measurement of the outcome? |  | NA / Favours experimental / Favours comparator / Towards null /Away from null / Unpredictable |

Domain 5: Risk of bias in selection of the reported result

| **Signalling questions** | **Comments** | **Response options** |
| --- | --- | --- |
| **5.1 Were the data that produced this result analysed in accordance with a pre-specified analysis plan that was finalized before unblinded outcome data were available for analysis?** | **5.1** The analysis appears aligned with pre-specified plans, though post-hoc analyses were conducted.  **Judgment:** PY | Y / PY / PN / N / NI |
| **Is the numerical result being assessed likely to have been selected, on the basis of the results, from...** |  |  |
| **5.2. ... multiple eligible outcome measurements (e.g. scales, definitions, time points) within the outcome domain?** | **5.2** There is no strong evidence of selective reporting across multiple measurements.  **Judgment:** PN | Y / PY / PN / N / NI |
| **5.3 ... multiple eligible analyses of the data?** | **5.3** Post-hoc subgroup analyses raise some concern about selective analysis.  **Judgment:** PY | Y / PY / PN / N / NI |
| **Risk-of-bias judgement** | **Some concerns** Comment: While the primary analysis appears pre-specified, post-hoc analyses introduce potential bias. | Low / High / Some concerns |
| Optional: What is the predicted direction of bias due to selection of the reported result? |  | NA / Favours experimental / Favours comparator / Towards null /Away from null / Unpredictable |

Overall risk of bias

| **Risk-of-bias judgement** | **Some concerns** Comment: The main concerns stem from potential differences in outcome assessment between groups and selective reporting/analysis in secondary outcomes. | Low / High / Some concerns |
| --- | --- | --- |
| Optional: What is the overall predicted direction of bias for this outcome? | Unpredictable. | NA / Favours experimental / Favours comparator / Towards null /Away from null / Unpredictable |


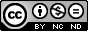


This work is licensed under a [Creative Commons Attribution-NonCommercial-NoDerivatives 4.0 International License](http://creativecommons.org/licenses/by-nc-nd/4.0/).
