# Supplementary material for: Impact of Nurse‐Driven Analgesia and Sedation Protocols on Medication Exposure and Withdrawal in Critically Ill Children: A Systematic Review
Source: Nurs Crit Care. 2025 May 29;30(3):e70051. doi: 10.1111/nicc.70051 (PMC12120589; doi:10.1111/nicc.70051)
Supplement: Supplementary file 2 — Document 1. Risk of bias assessment results for the RESTORE trial by Curley et al (2015). Document 2. Risk of bias assessment results for the cardiac‐RESTORE by Lincoln et al (2020). Document 3. Risk of bias assessment results for Magner et al (2020). Document 4. Risk of bias assessment results for Dreyfus et al (2017). Document 5. Risk of bias assessment results for Neunhoeffer et al (2015). Document 6. Risk of bias assessment results for Neunhoeffer et al (2017). Document 7. Risk of bias assessment results for Michel et al (2020). Document 8. Risk of bias assessment results for Gaillard‐Le Roux et al (2017). Document 9. Risk of bias assessment results for Larson and McKeever (2018). Document 10. Risk of bias assessment results for Hanser et al (2020). [file NICC-30-0-s002.zip › Supplementary document 2.docx]

The Risk Of Bias In Non-randomized Studies – of Interventions, Version 2 (ROBINS-I V2) assessment tool

(for follow-up studies)

**November 2024**


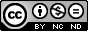


This work is licensed under a [Creative Commons Attribution-NonCommercial-NoDerivatives 4.0 International License](http://creativecommons.org/licenses/by-nc-nd/4.0/).

VERSION 2: LAUNCH VERSION, 22 November 2024

## Outline of ROBINS-I V2

ROBINS-I aims to assess the risk of bias in a specific result from an individual non-randomized study that examines the effect of an intervention on an outcome. This document describes the ROBINS-I V2 tool for **follow-up (cohort) studies**. Assessments should relate to risk of **material bias** rather than risk of any bias. Material bias should be interpreted as bias sufficient to cause an important change to the magnitude of the estimated effect, compared with the true value.

Before undertaking a ROBINS-I assessment (or series of assessments, e.g. in the context of a systematic review), users of the tool should specify the important confounding factors that are likely to influence the association between the intervention and the outcome (see section “At planning stage”).

The start point for an assessment of a specific study is to specify the result from the study that is being assessed for risk of bias. A ‘screening’ section then facilitates identification of results that are at “Critical risk of bias”, allowing the user to avoid a detailed assessment.

A key feature of the ROBINS-I approach is the specification, for each study, of the causal effect estimated by the result under consideration through specification of a hypothetical ‘target trial’. This is essential for assessment of risk of bias, because the causal effect defines the result that would be seen (other than the impact of sampling variation) in the absence of bias.

If multiple assessors will implement ROBINS-I independently, the *Preliminary considerations to plan the assessment* should be agreed between all assessors before each assessor works individually through evaluation of the confounding factors and bias domains.

ROBINS I includes seven domains of bias:

- Domain 1: Risk of bias due to confounding
- Domain 2: Risk of bias in classification of interventions
- Domain 3: Risk of bias in selection of participants into the study (or into the analysis)
- Domain 4: Risk of bias due to deviations from intended interventions
- Domain 5: Risk of bias due to missing data
- Domain 6: Risk of bias arising from measurement of the outcome
- Domain 7: Risk of bias in selection of the reported result

Each bias domain in ROBINS-I is addressed using a series of **signalling questions** that aim to gather important information about the study and the analysis being assessed. Most signalling questions have response options ‘Yes’, ‘Probably yes’, ‘Probably no’, ‘No’ and ‘No information’, with ‘Yes’ and ‘Probably yes’ having the same implications for risk of bias and similarly for ‘No’ and ‘Probably no’. Some questions have additional response options (a ‘weak’ and a ‘strong’ version of ‘Yes’ or ‘No’) to help discriminate between higher and lower risk of bias. After the relevant signalling questions have been completed, an algorithm maps the answers to the signalling questions onto a proposed judgement about **risk of bias** in the result that arises from this domain. The judgements and their broad interpretations are as follows.

| Judgement | Interpretation |
| --- | --- |
| *Low risk of bias** | There is little or no concern about bias with regard to this domain. |
| *Moderate risk of bias* | There is some concern about bias with regard to this domain, although it is not clear that there is an important risk of bias. |
| *Serious risk of bias* | The study has some important problems in this domain: characteristics of the study give rise to a serious risk of bias. |
| *Critical risk of bias* | The study is very problematic in this domain: characteristics of the study give rise to a critical risk of bias, such that and the result should generally be excluded from evidence syntheses. |

*For Domain 1 (Risk of bias due to confounding), this is referred to as “Low risk of bias (except for concerns about uncontrolled confounding)”, in which confounding is very well addressed but cannot be eliminated as a possibility. This is because a risk of bias due to uncontrolled confounding cannot be excluded in an observational study.

ROBINS-I is intended to provide a framework for making informed and reasonable judgements about risk of material bias in studies of the effects of intervention on outcome. On occasion, answers to the signalling questions may not yield an appropriate risk of bias judgement based on the algorithm. Therefore, suggested risk of bias judgements produced by the algorithms can be overridden, in which case justification should be provided. We aim for transparency and reasonableness rather than mechanistic adherence to every word of the tool’s contents.

Optionally, a **predicted direction of bias** may be selected, balancing the various issues addressed within the domain. Response options for this depend on the type of bias being addressed.

After completing all seven bias domains, an **overall judgement** is made for the risk of bias (and optionally for the predicted direction of any bias). The risk-of-bias Judgement is derived from the domain-level judgements using an algorithm. As for bias domain-level judgements, justification should be provided when the overall judgement suggested by the algorithm is overridden.

An online implementation of ROBINS-I V2 including automatic selection of relevant signalling questions and algorithm-derived risk-of-bias judgements is available via www.riskofbias.info.

# The ROBINS-I V2 tool

## At planning stage: list confounding factors

P1. List the important confounding factors relevant to all or most studies on this topic. Specify whether these are particular to specific intervention-outcome combinations.

| Guidance notes A confounding factor is a prognostic factor that predicts the interventions received. Important confounding factors are those that have the potential to introduce material bias into an estimated effect. Factors that are expected to have only very weak associations with the intervention or with the outcome, such that failure to account for them in the analysis will not have a material impact on the estimated effect of intervention on outcome, need not be considered here. Important confounding factors should be pre-specified at the planning stage, for example in the protocol of a systematic review that will include studies of the effects of interventions. The identification of potential confounding factors requires content knowledge and may usefully be informed by examination of relevant literature. Important confounding factors should be specified at the level of the broad research question (e.g. using a single list of confounding factors for a systematic review). This broad question may cover several specific interventions and/or outcomes. If confounding factors are specific to particular intervention-outcome combinations, then this should be stated. |
| --- |

|  |
| --- |

# For each study result: preliminary considerations

| Guidance notes The following questions should be answered only for the specific result that is being evaluated for the current ROBINS-I assessment.  In case of multiple alternative analyses being presented, it is important to specify the numeric result (e.g. RR = 1.52 (95% CI 0.83 to 2.77) and/or a reference (e.g. to a table, figure or paragraph) that uniquely defines the result being assessed.  Some characteristics of a study or a result may lead directly to the result being at critical risk of bias, and so make detailed risk-of-bias assessments unnecessary. A series of preliminary questions in this section aim to identify such situations.  Two preliminary questions are used to examine whether there is a need to examine time-varying confounding in the first domain of the tool (Bias due to confounding). If participants could switch between intervention groups then associations between intervention and outcome may be biased by time-varying confounding. This occurs when prognostic factors influence switches between intended interventions. For example, in a cohort study of the effect of antiretroviral therapy (ART) on rates of AIDS and death in people with HIV, follow-up time for each participant was split according to receipt of ART. Because CD4 counts during follow-up influenced the decision to start ART, CD4 count was a time-varying confounder.  The **target randomized trial specific to the study** is a hypothetical randomized trial, which need not be ethical or feasible, that compares the health effects of the same interventions, conducted with the same eligibility criteria as the non-randomized study. In general, such target trials will not use blinding of participants or of health professionals administering interventions.  **If multiple assessors will implement ROBINS-I independently, the questions in this section should be agreed between all assessors before each assessor works individually through the risk-of-bias assessment itself.** |
| --- |

## A. Specify the result being assessed for risk of bias

| 2BGuidance notes (Specifying the numerical result) A ROBINS-I assessment of risk of bias is specific to a particular study result. This is because different results from the same study may be at importantly different risks of bias (consider, for example, an unadjusted estimate of intervention effect compared with an estimate that is adjusted for numerous important confounding factors). Consequently, it may be necessary to undertake several ROBINS-I assessments of different results from the same study. If the study presents multiple alternative analyses, specify the numerical result (e.g. RR=1.52 (95% CI 0.83 to 2.77)) and/or a reference (e.g. to a table, figure or paragraph) that uniquely defines the result being assessed. |
| --- |

A1. Specify the numerical result being assessed

|  |
| --- |

A2. Provide further details about this result (for example, location in the study report, reason it was chosen) [optional]

|  |
| --- |

## B. Decide whether to proceed with a risk-of-bias assessment

| 3BGuidance notes (Whether to proceed with a risk-of-bias assessment) Some characteristics of a study or a result may lead directly to the result being at critical risk of bias, and so make detailed risk-of-bias assessments unnecessary. The questions in this section aim to identify such situations. |
| --- |

| **B1 Did the authors make any attempt to control for confounding?** | Confounding is a substantial problem in most non-randomized studies, and it is usually important to control for the important confounding factors. | Y / PY / PN / N |
| --- | --- | --- |
| **B2 If N/PN to B1: Is there sufficient potential for confounding that an unadjusted result should not be considered further?** | If there is sufficient potential for confounding that an unadjusted result should not be considered further, then the result is judged to be at ‘Critical risk of bias’. | Y / PY / PN / N |
| **B3 Was the method of measuring the outcome inappropriate?** | This question aims to identify methods of outcome measurement (data collection) that are unsuitable for the outcome they are intended to evaluate. This enables a rapid assessment that a result should be regarded as at ‘Critical risk of bias’.  The question does not aim to assess whether the choice of outcome being evaluated was *sensible* (e.g. because it is a surrogate or proxy for the main outcome of interest). In most circumstances, for pre-specified outcomes, the answer to this question will be ‘N’ or ‘PN’.  Answer ‘Y or ‘PY’ if the method of measuring the outcome is inappropriate, for example because:   1. important ranges of outcome values fall outside levels that are detectable using the measurement method; or 2. the measurement instrument has been demonstrated to have such poor reliability or validity that estimates of the relationship between intervention and the measured outcome are not useful. 3. The measurement method differed substantially between people in the intervention and comparator groups, so that differences between the groups are not interpretable. | Y / PY / PN / N |

**If the answer to either B2 or B3 is ‘Yes’ or ‘Probably yes’, the result should be considered to be at ‘Critical risk of bias’ and no further assessment is required.**

## C. Specify the analysis in the current study for which results are being assessed for risk of bias

Specify the outcome to which this result relates

|  |
| --- |

C1. Specify the participant group on which this result was based.

|  |
| --- |

*C2 to C3. Determine whether there is a need to consider time-varying confounding*

C2. Was the analysis based on splitting participants’ follow up time according to intervention received, or was follow-up censored when participants in one group switched to another group (e.g. when comparison group participants started the intervention)?

| □ | No | Use Variant A of Domain 1 |
| --- | --- | --- |
| □ | Yes | Proceed to next question |

C3. If **Y** to C2, were intervention discontinuations or switches likely to be related to factors that are predictive of the outcome?

| □ | No | Use Variant A of Domain 1 |
| --- | --- | --- |
| □ | Yes | Use Variant B of Domain 1 |

## D. Specify a (hypothetical) target randomized trial specific to the study

| Guidance notes Evaluations of risk of bias are facilitated by considering the non-randomized study as an attempt to emulate a pragmatic randomized trial, which we refer to as the **target trial**. The first part of a ROBINS-I assessment for a particular study is to specify a target trial - the hypothetical randomized trial whose results should be the same as those from the non-randomized study under consideration, in the absence of bias. Its key characteristics are the types of participant (including exclusion/inclusion criteria) and descriptions of the intervention strategy and comparator strategy. These issues were considered in more detail by Hernán (2016). Differences between the target trial for the individual non-randomized study and the generic research question of the review relate to issues of heterogeneity and/or generalizability rather than risk of bias.  Because it is hypothetical, ethics and feasibility need not be considered when specifying the target trial. For example there would be no objection to a target trial that compared individuals who did and did not start smoking, even though such a trial would be neither ethical nor feasible in practice.  Selection of a patient group that is eligible for a target trial may require detailed consideration, and lead to exclusion of many patients. For example, Magid et al (2010) studied the comparative effectiveness of ACE inhibitors compared to beta-blockers as second-line treatments for hypertension. From an initial cohort of 1.6m patients, they restricted the analysis population to (1) persons with incident hypertension, (2) who were initially treated with a thiazide agent, and (3) who had one of the two drugs of interest added as a second agent for uncontrolled hypertension, and (4) who did not have a contraindication to either drug. Their “comparative effectiveness” cohort included 15,540 individuals: less than 1% of the original cohort.  *A note on terminology*: Throughout ROBINS-I V2, we refer regularly to “intervention” and “comparator”. The comparator may be an alternative active intervention, a control condition or no intervention at all.  We sometimes refer to the “intervention strategy” and “comparator strategy”, because an intervention typically consists of a package of care or procedures, and may be implemented over a period of time rather than on a single occasion. Specification of the whole strategy of interest is particularly important when interest is in a ‘per protocol’ effect.  In non-randomized studies, assignment to the intervention or comparator is inferred from the recorded intervention for each participant. This is in contrast to randomized trials, in which participants are randomly assigned to the intervention or comparator. We refer to the participants assigned to each strategy as the “intervention group” and “comparator group”.  Hernán MA, Robins JM. Using big data to emulate a target trial when a randomized trial is not available. *American Journal of Epidemiology* 2016;183:758-64; doi:10.1093/aje/kwv254.  Magid DJ, Shetterly SM, Margolis KL, Tavel HM, O’Connor PJ, Selby JV, Ho PM. Comparative effectiveness of angiotensin-converting enzyme inhibitors versus beta-blocker as second-line therapy for hypertension. *Circulation: Cardiovascular Quality and Outcomes* 2010;3:453-458; doi:10.1161/CIRCOUTCOMES.110.940874. |
| --- |

| D1. Specify the participants and eligibility criteria |  |
| --- | --- |
| D2. Specify the intervention strategy |  |
| D3. Specify the comparator strategy |  |

## E. Decide on the effect of interest

E1. Is your aim for this study…?

| □ | to assess the intention-to-treat effect (the effect of *assignment to* an intervention strategy or comparator strategy) |
| --- | --- |
| □ | to assess a per-protocol effect (the effect of *adhering to* a specified intervention strategy or comparator strategy) |

E2. **If the aim is to assess a per-protocol effect**, briefly define the changes to the intervention or comparator strategies that will be considered to be protocol deviations and, optionally, those changes that will not be considered. For example, the protocol deviations considered could be: “Starting intervention among comparator group participants, while acceptable changes could be “stopping intervention because of intervention-related toxicities occur or disease progression” or “changes to intervention after the trial baseline”.

|  |
| --- |

# F. Information sources

| Guidance notes Evaluation of a study should be based on the maximum possible amount of available information. In addition to published papers describing a study’s methods and results, such information may be derived from the study protocol, unpublished reports or through correspondence with the study investigators. |
| --- |

Which of the following sources have you obtained to help you inform your risk of bias judgements (tick as many as apply)?

- Journal article(s)
- Study protocol
- Statistical analysis plan (SAP)
- Non-commercial registry record (e.g. ClinicalTrials.gov record)
- Company-owned registry record (e.g. GSK Clinical Study Register record)
- “Grey literature” (e.g. unpublished thesis)
- Conference abstract(s)
- Regulatory document (e.g. Clinical Study Report, Drug Approval Package)
- Individual participant data
- Research ethics application
- Grant database summary (e.g. NIH RePORTER, Research Councils UK Gateway to Research)
- Personal communication with investigator
- Personal communication with sponsor

Please specify any additional sources not listed above

|  |
| --- |

# Evaluation of confounding factors

Complete a row for each important confounding factor listed in advance (subsection (i) below); and either relevant to the setting of this particular study or identified by the study authors (subsection (ii)). **“Important” confounding factors are those for which, in the context of this study,** **adjustment is expected to lead to a meaningful change in the estimated effect of the intervention.**

| Guidance notes Confounding is of fundamental importance to the analysis and interpretation of non-randomized studies of the effect of interventions on outcomes. ROBINS-I addresses two types of confounding: baseline confounding and time-varying confounding. **Baseline confounding** occurs when one or more prognostic factors, present before the start of the intervention, predict intervention received. Appropriate methods to control for confounders measured at baseline include stratification, regression, matching, standardization, and inverse probability weighting. The analysis may control for individual variables or for estimated propensity scores (inverse probability weighting is based on a function of the propensity score).  **Time-varying confounding** needs to be considered in studies that partition follow-up time for individual participants according to intervention received.  We use the term **confounding factor** for each broad source of potential confounding. It may not be possible to measure a factor well, and we distinguish between the confounding factor and the **variables** used to measure it. These variables may be used, for example, as covariates in a regression analysis.  In the context of a particular study, variables need not be included in the analysis: (a) if they are not associated with the outcome, conditional on intervention received (noting that lack of a statistically significant association is not evidence of a lack of association); (b) if they are not associated with intervention; (c) if adjustment makes no or minimal difference to the estimated effect of intervention on outcome; (d) because the confounder was addressed in the study design, for example by restricting to individuals with the same value of the confounder; (e) because a negative control demonstrates that there was unlikely to have been confounding due to this variable or that uncontrolled confounding was likely to be minimal; or (f) because external evidence suggests that controlling for the variable is not necessary in the context of the study being assessed.  In some studies, researchers may include a very large set of potential confounding variables in an analysis without considering their associations with outcome and intervention. Users of ROBINS-I should focus on (i) the confounding factors they determined a priori to be important and (ii) other factors for which adjustment is expected to lead to an important change in the estimated effect of the intervention on the outcome in the context of the current study.  Users of ROBINS-I should evaluate the confounding factors that they prespecified as important for the intervention-outcome relationship under study. The tool also allows the user to evaluate a second list of any further confounding factors that are either relevant to the setting of this particular study or which the study authors identified as potentially important. It is likely that new ideas relating to confounding and other potential sources of bias will be identified after the drafting of the review protocol, and even after piloting data collection from studies selected for inclusion in the systematic review. For example, such issues may be identified because they are mentioned in the introduction and/or discussion of one or more papers. This could be addressed in practice by explicitly recording whether potential confounders or other sources of bias are mentioned in the paper. |
| --- |

| In very rare situations it is possible that no confounding factors are present, either because interventions received are known to be unrelated to any prognostic factors for the outcome of interest, or because no such prognostic factors exist. In such situations, the risk of bias due to confounding may be assessed as low.  The purpose of this preliminary assessment of confounding factors is to review the extent to which the result being assessed was controlled for confounding, considering both the prespecified confounding factors and any further confounding factors identified as important in the context of the study being assessed. This enables users of ROBINS-I to answer the signalling questions for the Domain 1 assessment (Risk of bias due to confounding). “Important” confounding factors are those for which, in the context of this study, adjustment is expected to lead to an important change in the estimated effect of the intervention.  The preliminary assessment consists of the following steps for each confounding factor.   - determine which variables (if any) were measured for the factor; - determine which of these variables were controlled for in the analysis; - for variables that were not controlled for, look for evidence that controlling for the variable was not necessary in this particular study; - determine whether the confounding factor was measured validly and reliably by the variables used to measure it (this is assessed at the level of the confounding factor rather than the level of the individual variables used to measure the factor); - determine the likely direction of bias if the analysis fails to adjust for this variable (alone).   The direction of bias, if the analysis fails to adjust for a particular variable (alone), will be that the effect estimate is biased *upwards* or biased *downwards*. For example, if older age predicts that a particular intervention is more likely to be received and the outcome is mortality, then this confounding would bias the estimated effect downwards: unless we adjust for age the intervention will appear more positively associated with higher mortality than it should. In the presence of *positive confounding* (the confounder is positively associated with both intervention and outcome, or negatively associated with both intervention and outcome), the bias will be upwards. In the presence of *negative confounding* (the confounder is positively associated with intervention and negatively associated with outcome, or vice versa), the bias will be downwards. |
| --- |

| **(i) Important confounding factors listed in advance** | | | | | | |
| --- | --- | --- | --- | --- | --- | --- |
| Confounding factor | Measured variable(s) for this factor, if any | Was this variable (or were these variables) controlled for in the analysis?  (Y / N) | If this confounding factor was controlled for, was it measured validly and reliably by this variable (or these variables)?*  (NA / Y / PY / PN / N / NI) | If this confounding factor was not controlled for, is there evidence that controlling for it was unnecessary?**  (NA / Y / PY / PN / N) | OPTIONAL: Is failure to adjust for this confounding factor expected to bias the effect estimate upwards or downwards? (Upward bias (overestimate the intervention effect) / Downward bias (underestimate the intervention effect) / No information or unpredictable) | Comments |
|  |  |  |  |  |  |  |
|  |  |  |  |  |  |  |

| **(ii) Additional important confounding factors relevant to the setting of this particular study, or identified by the study authors** | | | | | | |
| --- | --- | --- | --- | --- | --- | --- |
| Confounding factor | Measured variable(s) for this factor, if any | Was this variable (or were these variables) controlled for in the analysis?  (Y / N) | If this confounding factor was controlled for, was it measured validly and reliably by this variable (or these variables)?*  (NA / Y / PY / PN / N / NI) | If this confounding factor was not controlled for, is there evidence that controlling for it was unnecessary?**  (NA / Y / PY / PN / N) | OPTIONAL: Is failure to adjust for this confounding factor expected to bias the effect estimate upwards or downwards? (Upward bias (overestimate the intervention effect) / Downward bias (underestimate the intervention effect) / No information or unpredictable) | Comments |
|  |  |  |  |  |  |  |
|  |  |  |  |  |  |  |

* “Validity” refers to whether the confounding variable or variables accurately measure the confounding factor, while “reliability” refers to the precision of the measurement (more measurement error means less reliability).

** In the context of a particular study, variables need not be included in the analysis: (a) ) if they are measured validly and reliably and are not associated with the outcome, conditional on intervention (noting that lack of a statistically significant association is not evidence of a lack of association; (b) if they are measured validly and reliably and are not associated with intervention; (c) if they are measured validly and reliably and adjustment makes no or minimal difference to the estimated effect of the primary parameter; (d) because the confounder was addressed in the study design, for example by restricting to individuals with the same value of the confounder; (e) because a negative control demonstrates that there was unlikely to have been confounding due to this variable or that uncontrolled confounding was likely to be minimal; or (f) because external evidence suggests that controlling for the variable is not necessary in the context of the study being assessed”.

# Risk of bias assessment

Responses underlined in green are potential markers for low risk of bias, and responses in red are potential markers for a risk of bias. Where questions relate only to sign posts to other questions, no formatting is used.

Citation: Lincoln, P. A., Whelan, K., Hartwell, L. P., Gauvreau, K., Dodsen, B. L., Larovere, J. M., Thiagarajan, R. R., Hickey, P. A. and Curley, M. A. Q. (2020) Nurse-Implemented Goal-Directed Strategy to Improve Pain and Sedation Management in a Pediatric Cardiac ICU, Pediatric Critical Care Medicine, -07-28, 21 (12), pp. 1064. DOI: 10.1097/pcc.0000000000002505.

## 1. Bias due to confounding

| Guidance notes The questions in this domain focus on the confounding factors that were identified as important in the preliminary evaluation in section E.  We use the term uncontrolled confounding to refer to confounding that was not controlled by the design or analysis of the study – and is therefore likely to bias the estimated effect of intervention. This may arise because (i) confounding factors were not (or could not) be measured; (ii) variables used to measure confounding factors were insufficient to characterize the confounding factor; or (iii) variables that characterize the confounding factor were measured but not included in the analysis. |
| --- |

#### Domain 1, Variant A (only baseline confounding needs to be addressed – if N to C2, or Y to C2 and N to C3)

| **Signalling questions** | **Elaboration** | **Response** |
| --- | --- | --- |
| **1.1 Did the authors control for all the important confounding factors for which this was necessary?** | The study adjusted for **patient age and complexity using the Society of Thoracic Surgeons-European Association for Cardio-Thoracic Surgery (STAT) score** and performed **linear regression adjustments** **for mechanical ventilation duration, ICU length of stay, and hospital length of stay**. However, **some potential confounders, such as variability in clinical practice among healthcare providers, were not fully controlled.**  Response: PY | Y / PY / WN (no, but uncontrolled confounding was probably not substantial) / SN (no, and uncontrolled confounding was probably substantial) / NI |
| **1.2 If Y/PY/WN to 1.1: Were confounding factors that were controlled for (and for which control was necessary) measured validly and reliably by the variables available in this study?** | **Yes.** The authors used **validated risk stratification tools (e.g., STAT score) and well-established sedation and withdrawal assessment tools** (State Behavioural Scale, Withdrawal Assessment Tool-version 1). The **electronic medical record (EMR) system was used for data extraction, ensuring consistency and accuracy in measurements.**  Response: Y | NA / Y / PY / WN (no, but the extent of measurement error in confounding factors was probably not substantial) / SN (no, and the extent of measurement error in confounding factors was probably substantial) / NI |
| **1.3 If Y/PY/WN to 1.1: Did the authors control for any post-intervention variables that could have been affected by the intervention?** | The study focused on **pre-post intervention comparisons** without explicitly adjusting for post-intervention variables that may have influenced outcomes. For example, **nursing and physician behavior changes post-intervention** could have affected sedation practices and pain management, yet these were not separately accounted for in the analysis.  Response: NI | NA / Y / PY / PN / N / NI |
| **1.4. Did the use of negative controls, quantitative bias analysis, or other considerations, suggest serious unmeasured confounding?** | **No formal negative controls or quantitative bias analyses were reported.** While the study **did not report major unexpected variations in patient outcomes**, the lack of control for **provider practice variations and potential differences in patient care approaches across the time periods studied** suggests **possible unmeasured confounding**.  Response: PN | NA / Y / PY / PN / N |
| Risk of bias judgement | Based on the answers above, the **risk of bias due to confounding** is **low except for concerns about uncontrolled confounding factors**. **The authors made efforts to control for key confounders, but without the use of advanced techniques for addressing potential unmeasured confounding, some bias could still exist**. | Low (except for concerns about uncontrolled confounding) / Moderate / Serious / Critical |
| Optional: What is the predicted direction of bias due to confounding? |  | Upward bias (overestimate the effect) / Downward bias (underestimate the effect) / Unpredictable |

Algorithm for reaching default risk of bias judgement:


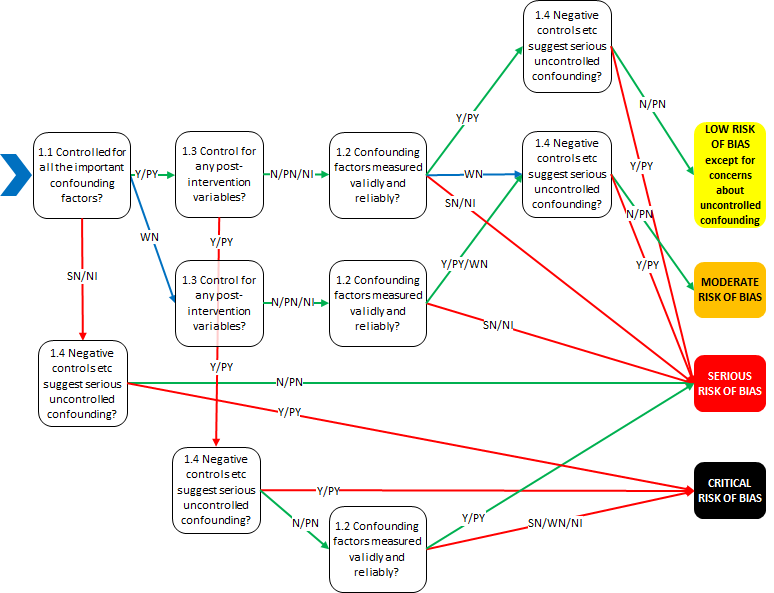


#### Domain 1, Variant B (the analysis was based on splitting participants’ follow up time according to intervention received, so both baseline and time-varying confounding need to be addressed – Y to C2 and Y to C3)

| **Signalling questions** | **Elaboration** | **Response** |
| --- | --- | --- |
| **1.1 Did the authors use an analysis method that was appropriate to control for time-varying as well as baseline confounding ?** |  | Y / PY / PN / N / NI |
| **1.2 If Y/PY to 1.1: Did the authors control for all the important baseline and time-varying confounding factors for which this was necessary?** |  | NA / Y / PY / WN (no, but uncontrolled confounding was probably not substantial) / SN (no, and uncontrolled confounding was probably substantial) / NI |
| **1.3 If Y/PY/WN to 1.2: Were confounding factors that were controlled for (and for which control was necessary) measured validly and reliably by the variables available in this study?** |  | NA / Y / PY / WN (no, but the extent of measurement error in confounding factors was probably not substantial) / SN (no, and the extent of measurement error in confounding factors was probably substantial) / NI |
| **1.4 If N/PN/NI to 1.1: Did the authors control for time-varying factors or other variables measured after the start of intervention?** |  | Y / PY / PN / N / NI |
| **1.5 Did the use of negative controls, or other considerations, suggest serious unmeasured confounding?** |  |  |
| Risk of bias judgement |  |  |
| Optional: What is the predicted direction of bias due to confounding? |  |  |

Algorithm for reaching default risk of bias judgement:


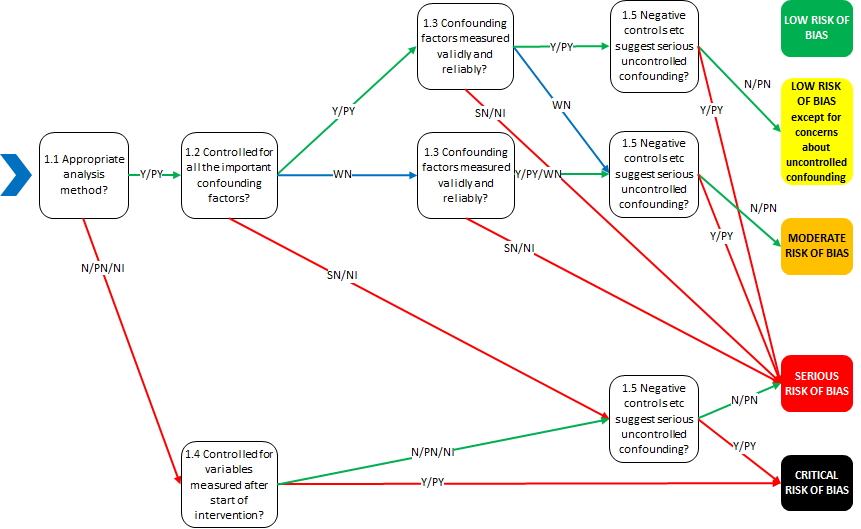


## 2. Bias in classification of interventions

| **Signalling questions** | **Elaboration** | **Response options** |
| --- | --- | --- |
| *Questions about immortal time bias arising from definition of intervention groups* |  |  |
| **2.1 Did assignment of participants to the intervention group or the comparator group rely on events or measurements that occurred after the start of follow up?** | The study used a **pre-post intervention design** where participants were assigned based on the time period they were admitted to the Pediatric Cardiac ICU. The intervention group consisted of **patients admitted after the implementation of the Cardiac-RESTORE protocol**, while the comparator group consisted of **patients admitted before the intervention**.  Response: N | Y / PY / PN / N / NI |
| **2.2 If Y/PY to 2.1: Were participants included in the comparator group until they fulfilled the definition of the intervention (or vice versa)?** |  | NA / SY (yes, and the impact was substantial) / WY (yes, but the impact was not substantial) / PN / N / NI |
| *Questions about differential misclassification* |  |  |
| **2.3 If N/PN to 2.1: Was all information used to classify intervention and comparator groups recorded at or before the time the interventions started?** | The classification of patients into intervention or comparator groups was based on **clear, pre-intervention criteria**. Data for patients in the **pre-intervention** and **post-intervention** time periods were recorded **before the intervention started**. This is important because it means that there was no influence of the outcomes on the classification of group assignment. **The patients were assigned based on the timeframes that had already been set in the protocol, prior to any outcomes being observed**.  Response: Y | NA / Y / PY / PN / N / NI |
| **2.4 Was classification of intervention status influenced by knowledge of the outcome or risk of the outcome?** | The classification of intervention status was not influenced by the outcome or the knowledge of it. The intervention (cardiac-RESTORE) and comparison (pre-intervention) groups were determined based on the **time periods** and **not influenced by individual outcomes**. This indicates a **relatively low risk of** **bias due to outcome knowledge** **when classifying intervention status**.  Response: N | SY (yes, and the impact was substantial) / WY (yes, but the impact was not substantial) / PN / N / NI |
| *Question about non-differential misclassification* |  |  |
| **2.5 If N/PN to 2.1 and WY/N/PN/NI 2.4: Was intervention status classified correctly for all, or nearly all, participants?** | Given the pre-defined nature of the intervention (cardiac-RESTORE) and the comparator (pre-intervention), **the classification was likely correct for** **nearly all participants**. Since there’s no mention of errors in the group assignment or misclassification, we can assume that the majority of participants were correctly assigned based on the defined time intervals, with minimal risk of misclassification.  Response: Y | NA / Y / PY / WN (no, but the impact was not substantial) / SN (no, and the impact was substantial) / NI |
| Risk of bias judgement | There is **low risk of bias** in the **assignment to groups** (both intervention and comparator groups) because:   - There was no immortal time bias, as participants were classified based on clear **time intervals**, which were defined prior to follow-up. - There was no influence of **knowledge of the outcome** on the classification of intervention status, reducing the risk of differential misclassification. | Low / Moderate / Serious / Critical |
| Optional: What is the predicted direction of bias in classification of interventions? |  | Favours intervention / Favours comparator / Towards null /Away from null / Unpredictable |

Algorithm for reaching default risk of bias judgement:


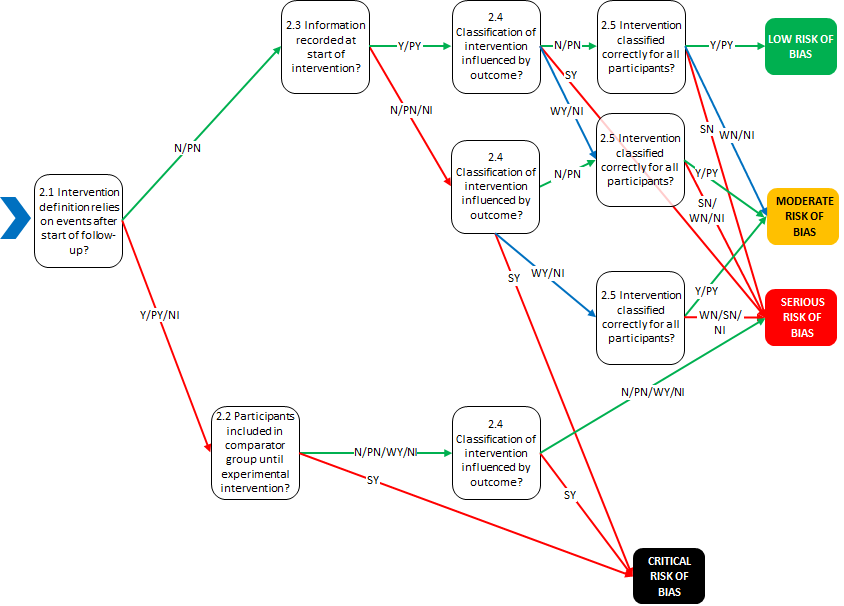


## 3. Bias in selection of participants into the study (or into the analysis)

In the target trial, start of follow up is the time at which participants meet eligibility criteria and are assigned to interventions. In answering the signalling questions for this domain, consider what is the start of follow up in the study under consideration, for both the intervention and comparison groups.

| **Signalling questions** | **Elaboration** | **Response options** |
| --- | --- | --- |
| *A. Questions about immortal time bias arising from definition of intervention groups* |  |  |
| **3.1 (=2.1) Did assignment of participants to the intervention group or the comparator group rely on events or measurements that occurred after the start of follow up?** | **The assignment of participants** to either the intervention group (post-intervention) or the comparator group (pre-intervention) **was based on** **pre-defined time intervals** and did not rely on any events or measurements that occurred after the start of follow-up.  Response: N | Y / PY / PN / N / NI |
| **3.2 If Y/PY to 3.1: Were participants excluded after the start of follow-up because they did not meet the definition of either the intervention or the comparator?** |  | NA / Y / PY / PN / N / NI |
| *B. Questions about prevalent user bias* |  |  |
| **3.3 Were start of follow up and start of intervention the same for most participants?** | For the majority of participants, the start of the intervention (cardiac-RESTORE) was well-defined, and its implementation began in the post-intervention periods, which aligned closely with the start of follow-up. Since it was a **time-based intervention**, the start of follow-up and the start of intervention should have been aligned for most participants.  Response: Y | NA / Y / PY / PN / N / NI |
| **3.4 If N/PN to 3.3: Is the effect of intervention expected to be constant over the time period studied?** |  | NA / Y / PY / PN / N / NI |
| *C. Questions about other types of selection bias* |  |  |
| **3.5 Was selection of participants into the study (or into the analysis) based on participant characteristics observed after the start of intervention (additional to the situations addressed in 3.1 and 3.3)?** | Participants were assigned to intervention or comparator groups **based on the timing of the intervention**, and the selection of participants did not depend on characteristics observed **after** the start of the intervention. The groups were not influenced by post-intervention characteristics.  Response: N | Y / PY / PN / N / NI |
| **3.6 If Y/PY to 3.5: Were the post-intervention variables that influenced selection likely to be associated with intervention?** |  | NA / Y / PY / PN / N / NI |
| **3.7 If Y/PY to 3.6: Were the post-intervention variables that influenced selection likely to be influenced by the outcome or a cause of the outcome?** |  | NA / Y / PY / PN / N / NI |
| *D. Questions about analysis, sensitivity analyses and severity of the problem* |  |  |
| **3.8 If Y/PY to 3.2, N/PN 3.4 or Y/PY to 3.7: Is it likely that the analysis corrected for all of the potential selection biases identified in 3.1-3.2, 3.3-3.4 or 3.5-3.7 above?** |  | NA / Y / PY / PN / N / NI |
| **3.9 If N/PN to 3.8: Did sensitivity analyses demonstrate that the likely impact of the potential selection biases identified in 3.1-3.2, 3.3-3.4 or 3.5-3.7 above was minimal?** |  | NA / Y / PY / PN / N / NI |
| **3.10 If N/PN to 3.9: Were potential selection biases identified in 3.1-3.2, 3.3-3.4 or 3.5-3.7 above sufficiently severe that the result should not be included in a quantitative synthesis?** |  | NA / Y / PY / PN / N / NI |
| Risk of bias judgement | The study has **low risk of selection bias**. The assignment to the intervention or comparator groups was based on **pre-intervention timing**, ensuring there was no reliance on post-intervention data for selection. The **prevalent user bias** and **immortal time bias** were minimized by clear group assignment and consistent follow-up periods. There was no **post-intervention selection** of participants or major influence of post-intervention characteristics. | Low / Moderate / Serious / Critical |
| Optional: What is the predicted direction of bias in selection of participants into the study? |  | Favours intervention / Favours comparator / Towards null /Away from null / Unpredictable |

Algorithm for reaching default risk of bias judgement


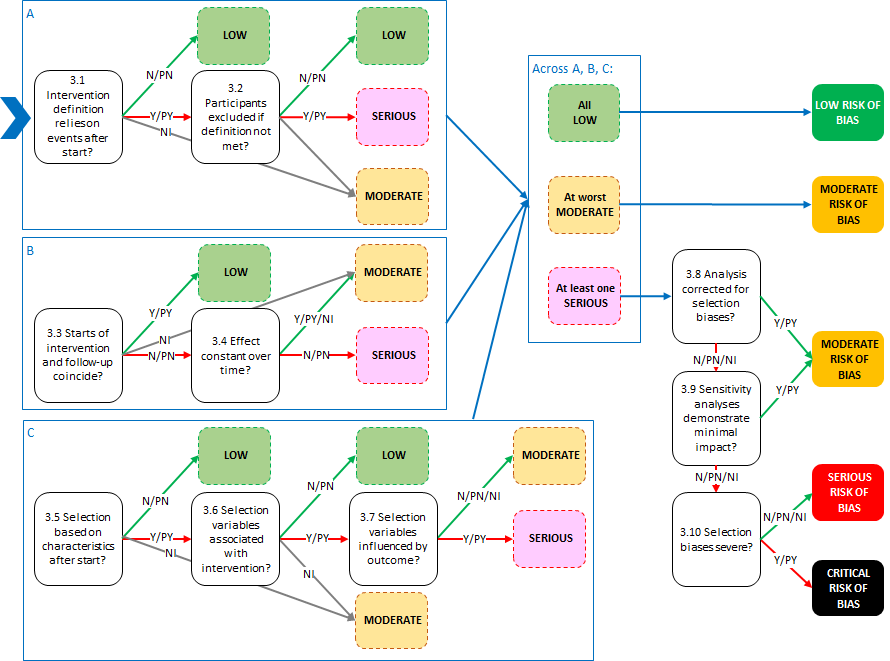


## 4. Bias due to deviations from intended interventions

#### Domain 4, Variant A: Effect of assignment to intervention

| **Signalling questions** | **Elaboration** | **Response options** |
| --- | --- | --- |
| **4.1 Was the study undertaken in an experimental context?** | This study was a **quality improvement project using a pre-post design**, not a randomized controlled trial (RCT) or experimental study. **Patients were not randomly assigned** to the intervention; instead, they were categorized based on whether they received care before or after the implementation of the Cardiac-RESTORE protocol.  Response: N | Y / PY / PN / N / NI |
| **4.2. If Y/PY to 4.1: Did participants deviate from the intended intervention as a result of the processes of recruiting and engaging them in the study?** |  | NA / Y / PY / PN / N / NI |
| **4.3. If Y/PY to 4.1: Did study personnel consciously or unconsciously undermine implementation of the intended interventions?** |  | NA / Y / PY / PN / N / NI |
| **4.4. If Y/PY/NI to 4.2 or 4.3: Were these deviations from intended intervention likely to have affected the outcome?** |  | NA / Y / PY / PN / N / NI |
| **4.5. Was an appropriate analysis used to estimate the effect of assignment to intervention?** | The study used **pre-post comparisons**, which can identify associations but **cannot fully account for secular trends or unmeasured confounders. Linear regression models were used to adjust for age and surgical complexity**, which is appropriate but does not eliminate **all possible biases**. **No randomized allocation or control for potential practice changes over time** beyond the intervention itself.  Response: PY | Y / PY / WN (no, but the impact was not substantial) / SN (no, and the impact was substantial) / NI |
| Risk of bias judgement | The study presents a **low risk of bias** in its methodology. Its strengths lie in the use of **validated assessment tools, a standardized intervention protocol, consistent data collection through electronic medical records (EMRs), and an adjusted statistical analysis that accounts for key confounders**. However, some limitations include **the potential influence of secular trends on the results, the absence of randomization, and possible variation in clinician adherence to the intervention**. Despite these limitations, the structured implementation and robust data collection minimize the likelihood of significant bias. | Low / Moderate / Serious / Critical |
| Optional: What is the predicted direction of bias in classification of interventions? |  | Favours intervention / Favours comparator / Towards null /Away from null / Unpredictable |

Algorithm for reaching default risk of bias judgement (effect of assignment to intervention)


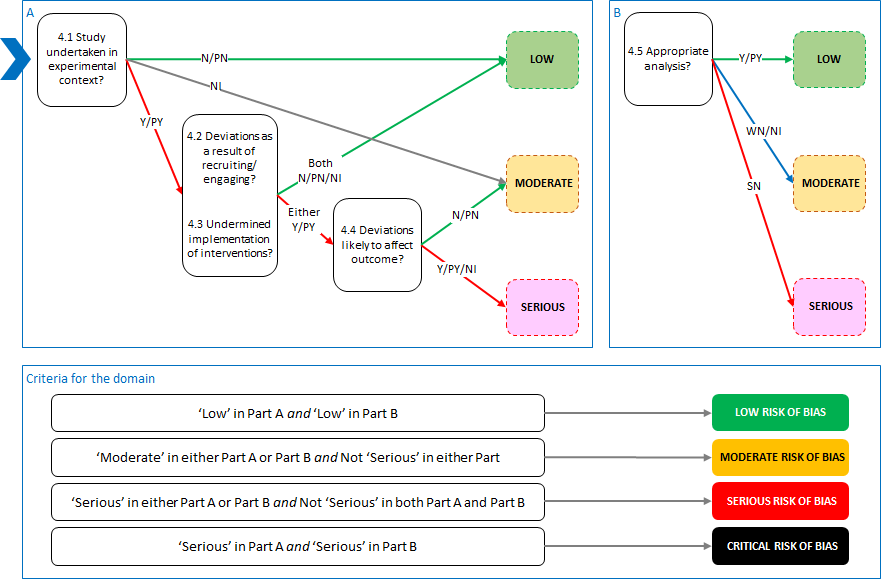


#### Domain 4, Variant B: Per-protocol effect (effect of adhering to intervention)

In answering the signalling questions below, consider the intervention strategies and protocol deviations specified in the preliminary considerations.

| **Signalling questions** | **Elaboration** | **Response options** |
| --- | --- | --- |
| **4.1 Did all or nearly all participants adhere to their assigned intervention strategy?** |  | Y / PY / PN / N / NI |
| **4.2. If N/PN/NI to 4.1: Were the protocol deviations likely to have affected the outcome?** |  | NA / Y / PY / PN / N / NI |
| **4.3 If Y/PY to 4.2: Was an appropriate analysis used to estimate the specified per-protocol effect, accounting for the specified protocol deviations?** |  | NA / Y / PY / PN / N / NI |
| Risk of bias judgement | See algorithm. | Low / Moderate / Serious / Critical |
| Optional: What is the predicted direction of bias in classification of interventions? |  | Favours intervention / Favours comparator / Towards null /Away from null / Unpredictable |

Algorithm for reaching default risk of bias judgement (effect of adhering to intervention)


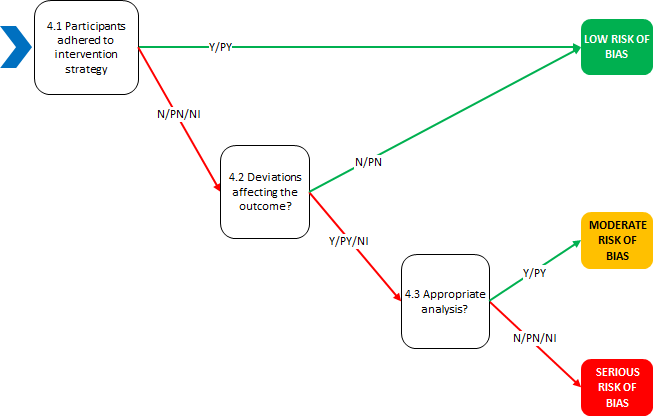


## 5. Bias due to missing data

| Guidance notes Missing outcome data may arise, among other reasons, through attrition (loss to follow up), missed appointments and incomplete data collection. Additionally, in non-randomized studies data may be missing for characteristics including interventions received and confounders.  A general rule for consideration of bias due to missing data is that we they should consider biases introduced by the missing data, compared with the effect estimate from an analysis in which all the data we intended to collect were available. Unfortunately, a single threshold for an acceptable proportion of missing data cannot meaningfully be defined. For example, a result based on 95% complete outcome data might be biased if the outcome was rare and if reasons for missing outcome data were strongly related to intervention group. Therefore, the potential for bias due to missing data should be assessed unless complete data on intervention status, the outcome and confounding variables were available all, or nearly all, participants.  Considerations of bias due to missing data depend on how the analysis accounted for the missing data. Different signalling questions should be answered depending on three types of analysis. The first is that a **complete case analysis**, restricted to participants with complete data on all the intervention, outcome and confounding variables, was performed. In this situation, an important consideration is whether missingness of individual participants from the analysis is related to the true value of the outcome for those participants. The second is that missing data were **imputed**, which means that estimated or assumed values were assigned to participants with missing data. Imputed data should not lead to bias if the data are ‘missing at random’ (see the elaboration for signalling question 5.8) and an appropriate imputation method is applied. Other types of analysis are addressed by a separate, general, signalling question. The final signalling question asks whether sensitivity analyses were performed that demonstrated that the impact of missing data is minimal. |
| --- |

| **Signalling questions** | **Elaboration** | **Response options** |
| --- | --- | --- |
| **5.1 Were complete data on intervention status available for all, or nearly all, participants?** | **The study collected data on intervention status across all time intervals**, with all participants categorized into either the pre-implementation or post-implementation groups. **There is no mention of significant missing data** **for intervention status**, so it's likely that complete data on intervention status were available for all or nearly all participants.  Response: Y | Y / PY / PN / N / NI |
| **5.2 Were complete data on the outcome available for all, or nearly all, participants?** | The study’s outcomes, such as medication dosing and risk-adjusted patient outcomes (e.g., duration of mechanical ventilation, length of PCICU and hospital stay), were systematically recorded. **There is no indication that any major outcomes were missing** **for participants**, suggesting that complete data on the outcome were available for most or all participants.  Response: Y | Y / PY / PN / N / NI |
| **5.3 Were complete data on important confounding variables available for all, or nearly all, participants?** | **The study controlled for several important confounding variables such as** **age, RACHS-1 categories**, and **STAT categories**. These variables were measured across all participants. The supplemental table (Table S1) suggests that these factors were accounted for in a standardized way, so **it is reasonable to assume that data on confounders were complete**.  Response: Y | Y / PY / PN / N / NI |
| **5.4 If N/PN/NI to 5.1, 5.2 or 5.3: Is the result based on a complete case analysis?** |  | NA / Y / PY / PN / N / NI |
| **5.5 If Y/PY/NI to 5.4: Was exclusion from the analysis because of missing data (in intervention, confounders or the outcome) likely to be related to the true value of the outcome?** |  | NA / Y / PY / PN / N / NI |
| **5.6 If Y/PY/NI to 5.5:** **Is the relationship between the outcome and missingness likely to be explained by the variables in the analysis model?** |  | NA / Y / PY / WN (No, but not leading to substantial bias) / SN (No, and bias is likely to be substantial) / NI |
| **5.7 If N/PN to 5.4: Was the analysis based on imputing missing values?** |  | NA / Y / PY / PN / NI |
| **5.8 If Y/PY to 5.7: Is it reasonable to assume that data were ‘missing at random’ (MAR) or ‘missing completely at random’ (MCAR)?** |  | NA / Y / PY / PN / N / NI |
| **5.9 If Y/PY to 5.8: Was imputation performed appropriately?** |  | NA / Y / PY / WN (no, but not leading to substantial bias) / SN (no, such that bias would not be substantially reduced) / NI |
| **5.10 If N/PN/NI to 5.7: Was an appropriate alternative method used to correct for bias due to missing data?** |  | NA / Y / PY / WN (no, but not leading to substantial bias) / SN (no, such that bias would not be substantially reduced) / NI |
| **5.11 If PN/N/NI to 5.1, 5.2 or 5.3 AND (Y/PY/NI to 5.5 OR (Y/PY to 5.8 AND WN/SN/NI to 5.9) OR WN/SN/NI to 5.10): Is there evidence that the result was not biased by missing data?** |  | NA / Y / PY / PN / N |
| Risk of bias judgement | Given that there was no missing data in the key variables (intervention status, outcome, or confounders), there is **low risk of bias** due to missing data. | Low / Moderate / Serious / Critical |
| Optional: What is the predicted direction of bias due to missing data? |  | Favours intervention / Favours comparator / Towards null /Away from null / Unpredictable |

Algorithm for reaching default risk of bias judgement:


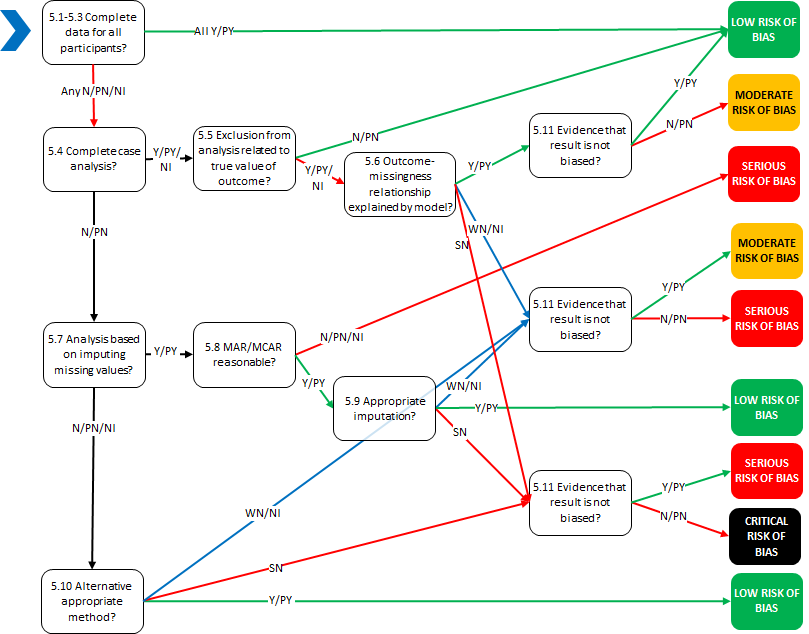


## 6. Bias in measurement of the outcome

| Guidance notes Bias may be introduced if outcomes are misclassified or measured with error. Misclassification or measurement error of outcomes may be non-differential or differential.  **Non-differential measurement error** is unrelated to the intervention received. It can be systematic (for example when measurement of blood pressure is consistently 5 units too high in every participant) – in which case it will not affect precision or cause bias; or it can be random (for example when measurement of blood pressure is sometimes too high and sometimes too low in a manner that does not depend on the intervention or the outcome) – in which case it will affect precision without causing bias.  **Differential measurement error** is measurement error related to intervention received. It will bias the intervention-outcome relationship. This is often referred to as detection bias. Examples of situations in which detection bias can arise are (i) if outcome assessors are aware of intervention received (particularly when the outcome is subjective); (ii) different methods (or intensities of observation) are used to assess outcomes of participants receiving different interventions; and (iii) measurement errors are related to intervention received (or to a confounder of the intervention-outcome relationship).  Blinding of outcome assessors aims to prevent systematic differences in measurements according to intervention received. However, blinding is frequently not possible or not performed for practical reasons. |
| --- |

| **Signalling questions** | **Elaboration** | **Response options** |
| --- | --- | --- |
| **6.1 Could measurement or ascertainment of the outcome have differed between intervention groups?** | The study employed standardized and objective measures for outcomes such as **ventilator duration**, **length of hospital stay**, and **PCICU duration**. These metrics are typically measured using consistent protocols and equipment, **making it unlikely that there was differential measurement between the intervention and comparator groups**. No evidence suggests that outcome ascertainment was affected by group assignment.  Response: N | Y / PY / PN / N / NI |
| **6.2 Were outcome assessors aware of the intervention received by study participants?** | **Outcome assessors were likely aware of the intervention group participants were assigned to**, as the study design involved different patient treatment regimens, which would likely require assessors to be informed to assess outcomes accurately (e.g., comparing post-implementation outcomes with pre-implementation ones). **This awareness could have influenced how outcomes were interpreted**, but the study does not provide clear information about any blinding of assessors.  Response: Y | Y / PY / PN / N / NI |
| **6.3 If Y/PY/NI to 6.2: Could assessment of the outcome have been influenced by knowledge of the intervention received?** | **Potentially.** Since nurses and physicians **actively participated in implementing the intervention and were aware of its goals**, their assessments of patient pain, sedation, and withdrawal symptoms could have been subtly influenced by an expectation of improvement. However, **the use of validated, standardized scales (e.g., the State Behavioral Scale and Withdrawal Assessment Tool) helps mitigate the risk of subjective bias in outcome assessment**.  Response: WY | NA / SY (yes, to a large extent) / WY (yes, to a small extent) / PN / N / NI |
| Risk of bias judgement | The risk of bias due to outcome measurement is **moderate**, as assessors were aware of the intervention status, which may have influenced their evaluations. However, the use of **standardized outcome measures, electronic medical record (EMR)-based data collection, and validated assessment tools** helps mitigate potential bias, ensuring a more reliable evaluation of patient outcomes. | Low / Moderate / Serious / Critical |
| Optional: What is the predicted direction of bias in measurement of outcomes? |  | Favours intervention / Favours comparator / Towards null /Away from null / Unpredictable |

Algorithm for reaching default risk of bias judgement:


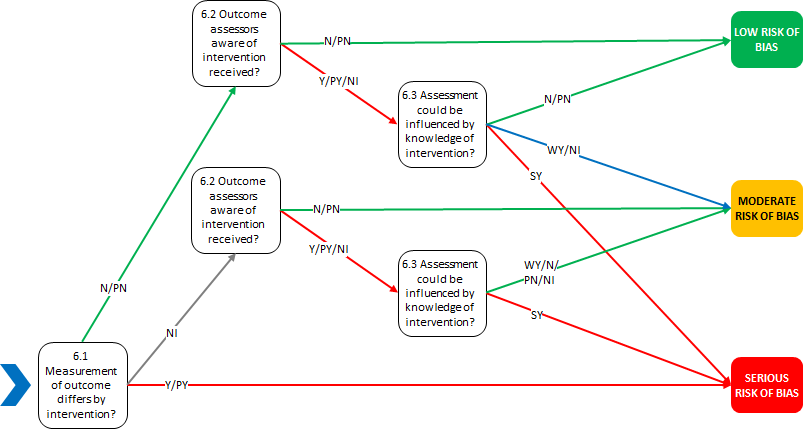


## 7. Bias in selection of the reported result

| Guidance notes Selective reporting can arise for both harms and benefits of an intervention, although the motivations (and direction of bias) underlying selective reporting of effect estimates for harms and benefits may differ. Selective reporting may arise, for example, from a desire for findings to be newsworthy (or sufficiently noteworthy to merit publication), or from commercial considerations, or from a desire to demonstrate that there is not evidence of a harmful effect of an intervention.  **Selective outcome reporting** occurs when the effect estimate for an outcome measurement was selected from among analyses of multiple outcome measurements for the outcome domain. Examples include: use of multiple measurement instruments (e.g. pain scales) and reporting only the most favourable result; reporting only the most favourable subscale (or a subset of subscales) for an instrument when measurements for other subscales were available; reporting only one or a subset of time points for which the outcome was measured.  **Selective analysis reporting** occurs when results are selected from effects estimated in multiple ways: e.g. carrying out analyses of both change scores and post-intervention scores adjusted for baseline; multiple analyses of a particular measurement with and without transformation; multiple analyses of a particular outcome with and without adjustment for potential confounders (or with adjustment for different sets of potential confounders); multiple analyses of a particular outcome with and without, or with different, methods to take account of missing data; a continuously scaled outcome converted to categorical data with different cut-points; multiple composite outcomes analysed for one outcome domain, but results were reported only for one (or a subset) of the composite outcomes. (Reporting an effect estimate for an unusual composite outcome might be evidence of such selective reporting.)  **Selection of a subgroup from a larger cohort**: The cohort for analysis may have been selected from a larger cohort for which data were available on the basis of a more interesting finding. Subgroups defined in unusual ways (e.g. an unusual classification of subgroups by dose or dose frequency) may provide evidence of such selective reporting.  The best evidence that results were not selectively reported is available if a pre-specified, publicly available analysis plan is available (e.g. from a link in a publication or from an online platform) and is in line with the reported results. Protocols for non-randomized studies are increasingly being registered, although there is inconsistency across platforms (Malmsiø et al, 2022). An analysis plan that is sufficiently detailed to permit full assessment of selective reporting may seldom be available for observational studies. In the absence of a protocol or analysis plan, clues can sometimes be gained by comparing Methods sections with Results sections.  Malmsiø D, Frost A, Hróbjartsson A. A scoping review finds that guides to authors of protocols for observational epidemiological studies varied highly in format and content. J Clin Epidemiol. 2022 Dec 20;154:156-166. doi: 10.1016/j.jclinepi.2022.12.012. |
| --- |

| **Signalling questions** | **Elaboration** | **Response options** |
| --- | --- | --- |
| **7.1 Was the result reported in accordance with an available, pre-determined analysis plan?** | The study followed a structured **pre-post design**, and the key outcomes (opioid and sedation use, pain scores, withdrawal symptoms) were reported consistently. However, the study did not explicitly state that it followed a **pre-registered analysis plan**, which introduces the possibility of selective reporting.  Response: PY | Y / PY / PN / N / NI |
| **Is the numerical result being assessed likely to have been selected, on the basis of the results, from...** |  |  |
| **7.2 ... multiple outcome *measurements* (e.g. scales, definitions, time points) within the outcome domain?** | The outcomes assessed (e.g., **opioid dosing**, **mechanical ventilation**, and **length of hospital stay**) appear to be directly aligned with the study’s primary objectives. It doesn’t seem that multiple outcome measurements within the same domain were selectively reported. **The analysis seems focused on these specific outcomes**, and no evidence suggests selective reporting based on the outcomes’ significance.  Response: N | Y / PY / PN / N / NI |
| **7.3 ... multiple *analyses* of the data?** | The study does not suggest that any results were **chosen after examining the data** for statistical significance or other reasons. The primary outcomes (such as opioid use, mechanical ventilation, and hospital length of stay) seem to have been part of the study design and were consistently analyzed across the four-time intervals, **without selective reporting based on statistical findings**. Therefore, it seems unlikely that the result was chosen post-hoc.  Response: N | Y / PY / PN / N / NI |
| **7.4 ... multiple *subgroups*?** | The study doesn’t seem to select or focus on results from **specific subgroups** after analyzing the full dataset. The outcomes appear to be evaluated across all participants in the time intervals without post-hoc subgrouping or selective reporting of certain groups based on their outcomes. No subgroup-specific data points or comparisons were explicitly highlighted as the main findings.  Response: N | Y / PY / PN / N / NI |
| Risk of bias judgement | The risk of **bias due to selective reporting** is **low**, as there is no evidence that the results were selectively chosen or manipulated based on data outcomes, subgroups, or multiple analyses. | Low / Moderate / Serious / Critical |
| Optional: What is the predicted direction of bias in selection of the reported result? |  | Favours intervention / Favours comparator / Towards null /Away from null / Unpredictable |

Algorithm for reaching default risk of bias judgement:


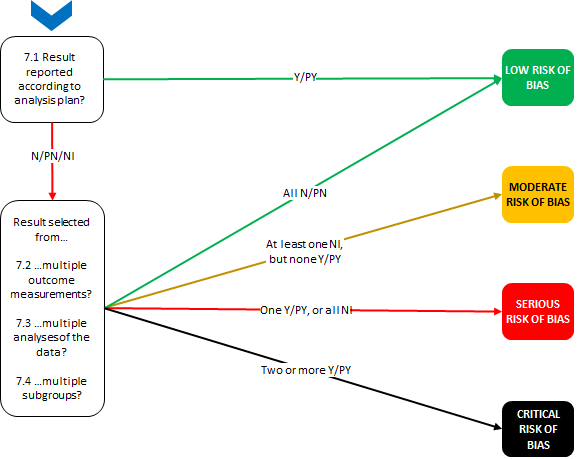
.

## Overall risk of bias

| Guidance notes ROBINS-I defaults to setting the overall risk of bias for a result to be equal to the risk-of-bias judgement for the domain with the greatest risk of bias. For example, if the ‘worst’ judgement across domains is of serious risk of bias, then the result would be judged as at serious risk of bias overall. However, the user may override this to judge the result to be at greater risk of bias if there are problems in several domains. For example, if several domains are assessed to be at serious risk of bias, and it is considered that these problems are likely to be compounded, then it may be reasonable to judge the result to be at critical risk of bias overall.  Predicting the direction of bias overall may be difficult. Risk-of-bias judgements for the individual domains might be used to inform the influence of that domain to the likely direction of bias overall. |
| --- |

| Overall risk of bias | The **overall risk of bias** in this study is **moderate**, with no serious concerns that would invalidate the findings. While some aspects, such as the lack of randomization, potential for observer bias, and possible selective reporting, introduce limitations, these are mitigated by the use of **standardized assessment tools, electronic medical records (EMRs), and statistical adjustments for key confounders**. | Low risk of bias except for concerns about uncontrolled confounding / Moderate risk / Serious risk / Critical risk |
| --- | --- | --- |
| What is the predicted direction of bias? | The **predicted direction of bias** is likely **in favor of the intervention**, as improvements in sedation and pain management may have been influenced by **secular trends, observer expectations, and potential selective reporting of favorable results**. However, given the structured implementation and consistent use of validated outcome measures, the overall findings remain **credible and informative**. | Upward bias (overestimate the effect) / Downward bias (underestimate the effect) / Favours intervention / Favours comparator / Towards null /Away from null / Unpredictable |

Algorithm for reaching overall risk of bias judgement:

| Judgement | Interpretation | How reached |
| --- | --- | --- |
| *Low risk of bias except for concerns about uncontrolled confounding* | There is the possibility of uncontrolled confounding that has not been controlled for (given the observational nature of the study), but otherwise little or no concern about bias in the result | *Low risk of bias except for concerns about uncontrolled confounding* in Domain 1 and *Low risk of bias* in all other domains |
| *Moderate risk of bias* | There is some concern about bias in the result, although it is not clear that there is an important risk of bias | At least one domain is at *Moderate risk of bias*, but no domains are at *Serious risk of bias* or *Critical risk of bias* |
| *Serious risk of bias* | The study has some important problems: characteristics of the study give rise to a serious risk of bias in the result | At least one domain is at *Serious risk of bias*, but no domains are at *Critical risk of bias*  OR  Several domains are at *Moderate*, leading to an additive judgement of *Serious risk of bias* |
| *Critical risk of bias* | The study is very problematic: characteristics of the study give rise to a critical of bias in the result, such that the result should generally be excluded from evidence syntheses. | At least one domain is at *Critical risk of bias*  OR  Several domains are at *Serious risk of bias*, leading to an additive judgement of *Crticial risk of bias* |


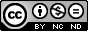


This work is licensed under a [Creative Commons Attribution-NonCommercial-NoDerivatives 4.0 International License](http://creativecommons.org/licenses/by-nc-nd/4.0/).
